# Molecular Epidemiology and Phyloevolutionary Analysis of Porcine Parvoviruses (PPV1 through PPV7) Detected in Replacement Gilts from Colombia

**DOI:** 10.3390/ijms251910354

**Published:** 2024-09-26

**Authors:** Diana S. Vargas-Bermudez, Bruno Aschidamini Prandi, Ueric José Borges de Souza, Ricardo Durães-Carvalho, José Darío Mogollón, Fabrício Souza Campos, Paulo Michel Roehe, Jairo Jaime

**Affiliations:** 1 Universidad Nacional de Colombia, Sede Bogotá, Facultad de Medicina Veterinaria y de Zootecnia, Departamento de Salud Animal, Centro de Investigación en Infectología e Inmunología Veterinaria–CI3V, Carrera 30 No. 45-03, Bogotá DC 111321, Colombia; dsvargasb@unal.edu.co (D.S.V.-B.); josedmogollon@yahoo.es (J.D.M.); 2 Virology Laboratory, Department of Microbiology, Immunology, and Parasitology, Institute of Basic Health Sciences, Federal University of Rio Grande do Sul, Porto Alegre 90050-170, Brazil; aschidamini.b@gmail.com (B.A.P.); camposvet@gmail.com (F.S.C.); proehe@gmail.com (P.M.R.); 3 Bioinformatics and Biotechnology Laboratory, Campus of Gurupi, Federal University of Tocantins, Gurupi 77410-570, Brazil; uericjose@gmail.com; 4 Department of Microbiology, Immunology and Parasitology, São Paulo School of Medicine, Federal University of São Paulo (UNIFESP), São Paulo 04039-032, Brazil; rdcarval@gmail.com; 5 Post-Graduate Program in Structural and Functional Biology, Department of Morphology and Genetics, UNIFESP, São Paulo 04039-032, Brazil

**Keywords:** porcine parvoviruses (PPVs), novel PPVs (nPPVs), genetic characterization, phylogenetic classification, swine herds, emerging pathogens

## Abstract

Eight porcine parvovirus (PPV) species, designated as PPV1 through PPV8, have been identified in swine. Despite their similarities, knowledge about their distribution and genetic differences remains limited, resulting in a gap in the genetic classification of these viruses. In this study, we conducted a comprehensive analysis using PPV1 to PPV7 genome sequences from Colombia and others available in the GenBank database to propose a classification scheme for all PPVs. Sera from 234 gilts aged 180 to 200 days were collected from 40 herds in Colombia. Individual detection of each PPV (PPV1 through PPV7) was performed using end-point PCR. Complete nucleotide (nt) sequencing was performed on the PPV1 viral protein (VP), and near-complete genome (NCG) sequencing was carried out for novel porcine parvoviruses (nPPVs) (PPV2 through PPV7). Phylogenetic analyses were conducted by comparing PPV1-VP sequences to 94 available sequences and nPPVs with 565 NCG, 846 nPPV-VP, and 667 nPPV–nonstructural protein (NS) sequences. Bayesian phylogenetic analysis was used to estimate substitution rates and the time to the most recent common ancestor for each PPV. The highest prevalence was detected for PPV3 (40.1%), followed by PPV5 (20.5%), PPV6 (17%), PPV1 (14.5%), PPV2 (9.8%), PPV4 (4.2%), and PPV7 (1.3%). Notably, all tested sera were negative for PPV8 genomes. An analysis of the PPV1-VP sequences revealed two main clades (PPV1-I and PPV1-II), with the sequences recovered in this study grouped in the PPV1-II clade. Comparative analysis showed significant genetic distances for PPV2 to PPV7 at the NCG (>6.5%), NS (>6.3%), and VP (>7.5%) regions, particularly when compared to equivalent regions of PPV genomes recovered worldwide. This study highlights the endemic circulation of nPPVs in Colombian pig herds, specifically among gilts. Additionally, it contributes to the phylogenetic classification and evolutionary studies of these viruses. The proposed method aims to categorize and divide subtypes based on current knowledge and the genomes available in databanks.

## 1. Introduction

During this century, with advancements in sequencing technologies and metagenomic analyses, novel species belonging to the *Parvoviridae* family have been identified [1]. Within the swine population, eight porcine parvovirus (PPV) species, designated as PPV1 through PPV8, have been documented and classified into various subfamilies and viral genera [2]. PPV2 through PPV8 are collectively referred to as novel PPVs (nPPVs) [3]. Seven of the eight PPVs belong to the *Parvovirinae* subfamily (PPV1 to PPV6 and PPV8), while PPV7 is classified within the *Hamaparvovirinae* subfamily [1].

A brief overview of each PPV reveals that PPV1 was first reported in the 1960s and is considered the earliest PPV. It is recognized as the primary etiological agent responsible for the porcine reproductive failure syndrome known as SMEDI (stillbirth, mummification, embryonic death, and infertility) [4]. The phylogenetic classification of PPV1 has been based on the high variability in the PPV1-VP sequence. Initially, two lineages (G1 and G2) were proposed [5], then between six and seven clusters (A to H) [6], subsequently four lineages (1 to 4) [7], and more recently, four genotypes (PPV1a to d) [8]. PPV2 was initially detected in Myanmar in 2001 [9] and subsequently identified across all continents [10,11,12,13]. Its presence in the lung tissue of pigs suffering from respiratory failure has led to its proposed role as a potential primary pathogen in the porcine respiratory disease complex (PRDC) [14,15]. Additional studies have suggested PPV2 as a possible participant in porcine reproductive failure (PRF) [16]. Initial phylogenetic classifications, based on incomplete nucleotide (nt) sequences of the PPV2-viral protein (VP) gene, resulted in its categorization into seven clades [17]. Subsequent analysis of complete PPV2-VP nt sequences have led to a proposed classification into two distinct clades, designated A and B [18,19]. PPV3 was first identified in Hong Kong in 2010 from swine samples collected at slaughterhouses [20] and has since been detected on multiple continents [18,21,22,23,24]. Sequencing of the complete PPV3-VP gene resulted in a proposed classification into four distinct groups (A to D) [18]. Recent studies focusing on the partial PPV3-VP1 gene (330bp) sequence suggest a classification into groups 1, 2, and 3 [25]. Furthermore, analysis of the coding sequence for the nonstructural protein 1 (NS1) revealed greater variability compared to the PPV3-VP sequence [26]. PPV4 was first reported in 2010 from porcine lung samples in the United States (USA) [27] and has subsequently been detected in various countries across Europe [22,23,28], Asia [29], Africa [12,30], and North and South America [31,32]. Its classification has been established based on complete PPV4-VP nt sequences, with two distinct groups (1 and 2) proposed [18,19,25]. Moving on to PPV5, it was initially identified in the USA in 2013 from porcine lung samples [33] and has since been detected in China [34], Republic of Korea [29], Poland [23], Mexico [32], and Brazil [35]. Based on the complete PPV5-VP nt sequence, PPV5 has been classified into two phylogenetic groups (A and B) [36]. PPV6 was first reported in China in 2014 from both abortions and stillbirths on herds experiencing PRF, as well as from healthy animals [37]. Subsequent reports have identified PPV6 in the USA and Mexico [38], Spain [39], Poland [40], Republic of Korea [29], and Brazil [35]. Analysis of complete PPV6-VP nt sequences has led to a proposed classification divided into three clades (I to III) [29].

PPV7 was discovered in 2016 in the USA through metagenomic analyses of stool samples [41] and has subsequently been reported in Asia [42], Europe [43], and South America [44]. A notable characteristic of PPV7 is its high variability at the VP (Cap) protein level, with strains exhibiting insertions of five amino acids (aa), corresponding to nt from 1410 to 1425 in the VP gene sequence, resulting in Cap length variations ranging from 470 to 475 aa [44]. Initial analysis of the PPV7 nt near-complete genome (NCG), excluding the VP insertion, suggested two clades (1 and 2) [42]. Consequently, another classification for PPV7 has been proposed, dividing it into two groups based on the presence or absence of this insertion [44], with more recent analyses indicating six clades (a to f) [45]. PPV8, the most recently reported PPV in 2022, was initially identified in the lungs of pigs with fever and respiratory signs in China [46]. By May 2024, this virus had not been reported outside of China. However, it is anticipated to be noted in Europe (Hungary and Slovakia) [47] and the Americas (Colombia) [48]. In addition to domestic pigs, PPV2 through PPV7 have been identified in wild boars [18,21,30,49,50], suggesting potential intraspecies and interspecies transmission within the swine population [18]. Furthermore, the evolutionary rates of parvoviruses are comparable to those of ssRNA viruses [51], a trend that may be influenced by coinfections and recombination events between PPVs. These occurrences could play a critical role in the natural evolution of PPVs [22,52], underscoring the significance of our research. Table 1 summarizes the information established so far on PPVs.

A consensus regarding the phylogenetic classification of nPPVs has yet to be established, and knowledge about their evolutionary dynamics remains limited. This is attributed to various factors, including the paucity of sequences deposited in databases, restricted circulation of these viruses, and the limited number of laboratories conducting diagnoses and genetic characterization. Given these challenges, the present study pursued two primary objectives: firstly, the identification of PPVs (PPV1 to PPV8) in replacement gilts and phyloevolutionary characterization of nPPVs (PPV2 to PPV7) detected on swine herds in Colombia. Secondly, based on the results obtained in the initial objective, we propose a new consensus classification for nPPVs (PPV2 to PPV7), utilizing our sequences and those available in the GenBank database.

## 2. Results

### 2.1. Detection Rates of PPVs in Replacement Gilts

This study detected PPV1 and nPPVs (PPV2 through PPV7) in the sera of 234 replacement gilts. Among these, PPV3 exhibited the highest prevalence, detected in 40.1% of the gilts, followed by PPV5 in 20.5%, PPV6 in 17%, PPV1 in 14.5%, PPV2 in 9.8%, PPV4 in 4.2%, and PPV7 in 1.3% (Table 2). At the herd level, the most prevalent PPVs were PPV3 (67.5%), followed by PPV5 (40%), PPV6 (32.5%), PPV2 (22.5%), PPV1 (17.5%), PPV4 (15%), and PPV7 (5%). Notably, all sera tested negatively for PPV8.

**Figure 1:**
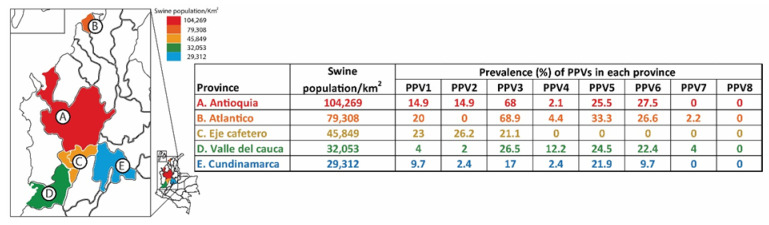
Map of Colombia highlighting the five major swine-producing provinces and the swine density of each one. From these provinces, 40 herds (8 per province) were randomly selected, and serum samples from 234 gilts were collected. A. Antioquia (*n* = 47); B. Cundinamarca (*n* = 41); C. Atlántico (*n* = 45), D. Eje Cafetero (*n* = 52), and E. Valle del Cauca (*n* = 49). The percentage of porcine parvoviruses (PPV1 through PPV8) detected in each province is indicated. The map was created using the National Department of Statistics of Colombia (DANE) database (https://geoportal.dane.gov.co/acerca-del-geoportal/acerca/#gsc.tab=0) (accessed on 12 April 2024) and adapted using the QGIS software version 3.34.5 available online: https://qgis.org/es/site/ (accessed on 12 April 2024).

Regarding geographical distribution, PPV1 and PPV3 were detected in all five provinces studied (100% prevalence). PPV2, PPV4, PPV5, and PPV6 were found in four provinces (80%) and PPV7 in two (40%). The Valle del Cauca province was the only one where all PPVs (PPV1 through PPV7), except PPV8, were identified (Figure 1).

Of the 234 sera analyzed, 30% (70/234) tested negatively for all PPVs. Among positive samples, PPVs were identified in both monoinfections and coinfections involving up to four distinct PPVs. Monoinfections accounted for 41.4% (97/234) of cases, with PPV3 being the most prevalent at 37% (36/97), followed by PPV5 at 22.6% (22/97). Coinfections were detected in various combinations: dual infections were found in 21% (49/234) of samples.

The most common dual infection involved was PPV3/PPV6 in 32.6% (16/49) of cases, followed by PPV3/PPV5 at 28.6% (14/49) (Table 3). Triple coinfections were detected in 6.4% (15/234) of the total samples. The most prevalent were PPV1/PPV3/PPV6 at 26.6% (4/15), PPV2/PPV3/PPV5 at 20% (3/15), PPV1/PPV3/PPV4 (2/15), and PPV1/PPV3/PPV5 (2/15), each at 13.3%. Other triple coinfections observed once each included PPV2/PPV3/PPV4, PPV3/PPV5/PPV6, PPV4/PPV5/PPV6, and PPV4/PPV6/PPV7. Quadruple coinfections were also identified in 1.28% (3/234) of samples, corresponding to PPV1/PPV3/PPV6/PPV7, PPV3/PPV4/PPV5/PPV6, and PPV3/PPV4/PPV6/PPV7, each occurring in one sample. Table S1 provides a comprehensive summary of the triple and quadruple coinfections identified in this study. Noteworthy, coinfections were detected across all five geographical provinces as in all herds evaluated, while PPV7 was only detected in triple and quadruple coinfections.

### 2.2. Phylogenetic and Evolutionary Rate Analysis of PPV1 and nPPVs

For PPV1, two complete PPV1-VP nt sequences recovered in this study (GenBank accession numbers PP921711 and PP921712) were compared to 94 reference strains of PPV1-VP (Table S2). Phylogenetic analysis revealed that PPV1-VP sequences clustered into two main clades (PPV1-I and PPV1-II), each with distinct subclades. PPV1 clade I comprises strains that have been circulating globally for over 60 years. PPV1 subclade I includes reference strains such as the attenuated strain NADL-2 (KF913346), and subclade II includes the Porcilis vaccine strain (ON014618) and virulent strain NADL8 (ON014603).

**Figure 2:**
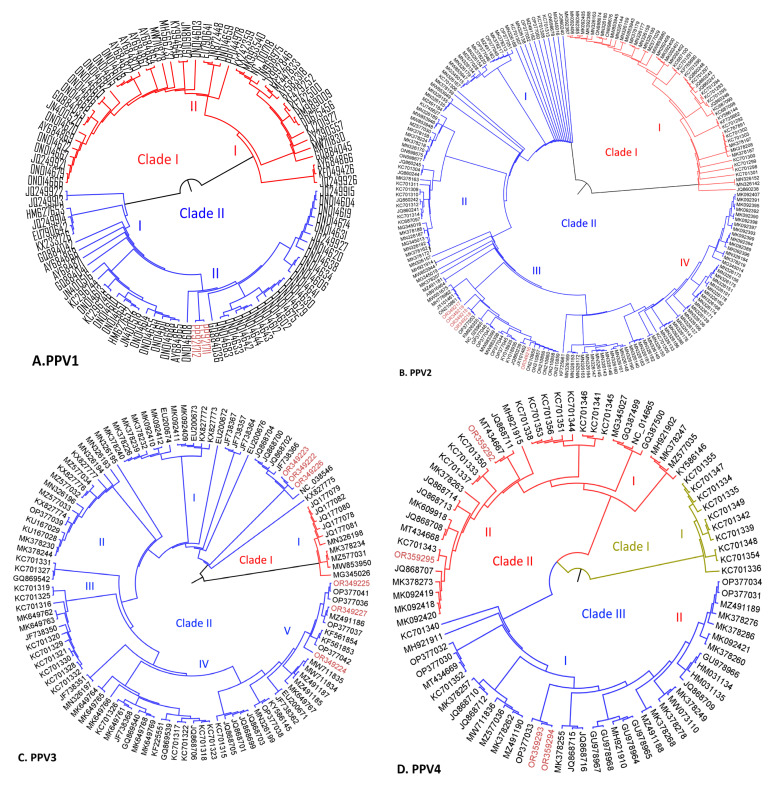

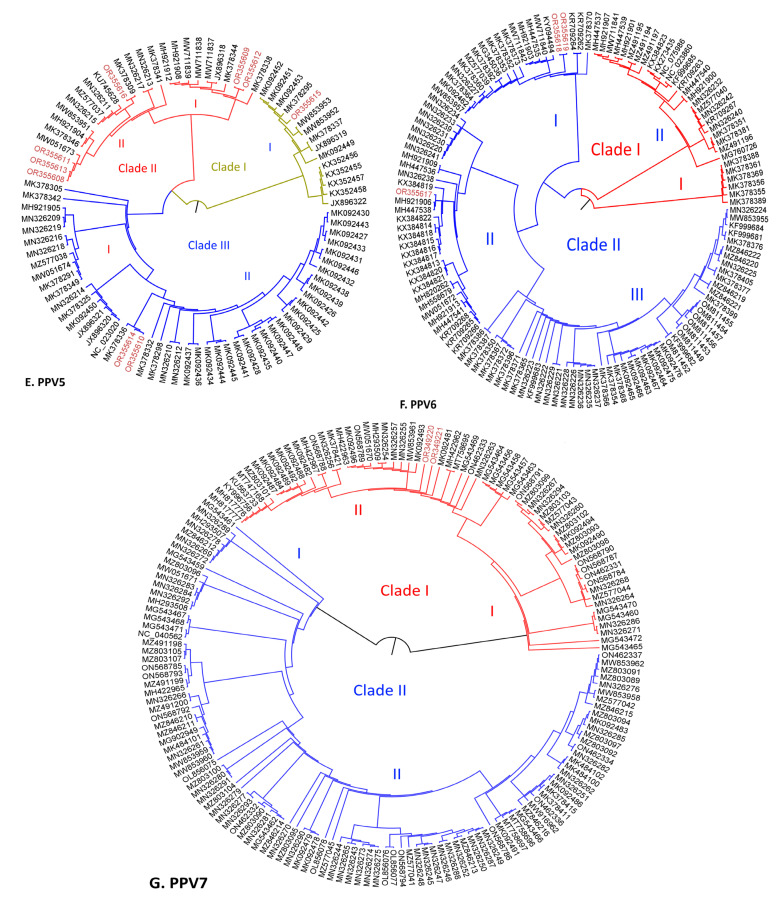
Maximum likelihood phylogenetic trees of PPVs (PPV1 through PPV7) estimated based on the alignment of the nucleotide sequences encoding VP. (**A**) PPV1, (**B**) PPV2, (**C**) PPV3, (**D**) PPV4, (**E**) PPV5, (**F**) PPV6, and (**G**) PPV7. Colombian sequences are highlighted in red.

PPV1 clade II primarily encompasses sequences reported from Europe since the beginning of this decade. PPV1-I subclade I includes the hypervirulent strains (27a-like) (AY684871) from Germany and some sequences from China, while the two sequences from Colombia were positioned in the PPV1-II subclade II, showing high identity with sequences from France and Denmark identified in the same year, 2021 (ON014608 and ON014683, respectively) (Figure 2A). The aa residue at position 586 was identified as the primary determinant clade differentiation. Clade I is characterized by the presence of S or P at this position; whereas, clade II exhibits A or T. Subsequent analyses of the PPV1-VP aa sequences revealed that the two Colombian sequences share identical variations with those previously reported in hypervirulent 27a-like strains, specifically Q378E, A564S, E569Q, and P586T substitutions. To date, various phylogenetic studies of PPV1 have not reached a consensus on classification. Table 4 presents a comparative overview between different PPV1 classification schemes proposed in previous investigations and the current study.

To propose a consensus classification for each nPPV (PPV2 through PPV7), we analyzed three sets of sequences: complete nt sequences of nPPV-VP, complete nt sequences of nPPV-NS, and nPPV–near-complete genome (NCG). This approach was supported by the abundance of these three sequence types in the GenBank database. Table 5 summarizes the nt and aa sequence identities between the nPPV strains reported in this study and those deposited in the GenBank database.

A comprehensive analysis comparing the sequences of the nPPVs revealed that PPV2 and PPV7 exhibited the most significant genetic distances at the level of NCG (>6.5%), NS (>6.3%), and VP (>7.5%), particularly when compared to sequences from around the world (including the Colombian sequences). Among the nPPVs, a higher genetic distance was observed at the VP level (5.5–9.5%), except for PPV3, which displayed a greater genetic distance at the NS level (4%) compared to the VP level (3%). PPV7 exhibited the greatest genetic diversity across all three sequence types (NCG, VP, and NS) evaluated.

**Figure 3:**
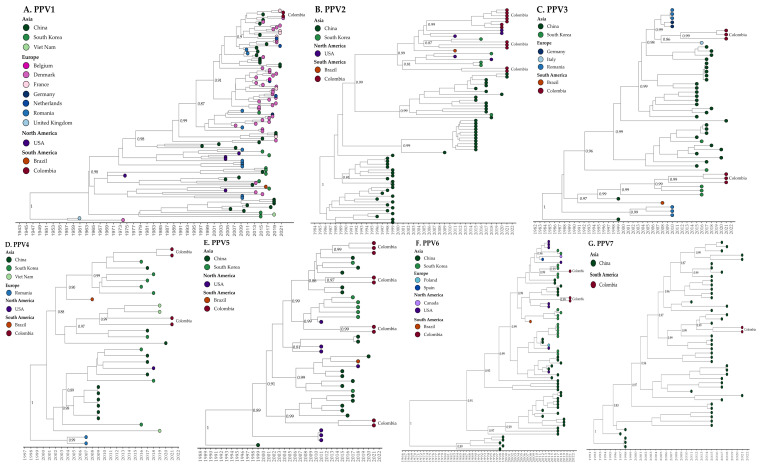
Time-scaled phylogenetic trees of nPPVs’ complete and near-complete genome sequences available on GenBank. Different sampling locations are represented by colors, as indicated in the legend on the left side of the tree. (**A**) PPV1 (114 sequences), (**B**) PPV2 (74 sequences), (**C**) PPV3 (52 sequences), (**D**) PPV4 (32 sequences), (**E**) PPV5 (42 sequences), (**F**) PPV6 (71 sequences), and (**G**) PPV7 (49 sequences).

Regarding the evolution rate and phylogeny for each PPV, the nt substitution rates and times to common ancestry are presented in Table 6. The estimated evolutionary rate for PPV1 (114 VP sequences, Table S3) was 1.34 *×* 10^−4^ nt substitutions per site per year (nspy), with a 95% highest-posterior-density interval (HDP) ranging from 5.30 × 10^−5^ to 1.08 × 10^−4^ nspy. The time of the most recent common ancestor (TMRCA) for the phylogenetic evolutionary tree was estimated to be around October 1946, utilizing the most recently analyzed sequence (2021), with a 95% HPDI ranging from October 1928 to December 1962 (Figure 3A).

Table 7 presents a comparative overview between different nPPV classification schemes proposed in previous investigations and the current study. The previous ones were chosen based on having evaluated at least two different PPVs together with the proposal of their phylogenetic classification. The estimated evolutionary rate for PPV2 (74 NCG and complete genome, Table S4) was 5.74 × 10^−4^ nspy, with a 95% highest-posterior-density interval (HPDI). The time of the most recent common ancestor (TMRCA) for the phylogenetic evolutionary tree was estimated to be around December 1984, utilizing the most recently analyzed sequence (2021), with a 95% HPDI ranging from May 1980 to July 1988 (Figure 3B). For the phylogenetic classification of PPV2, PPV2-NCG (145 nt sequences) and PPV2-VP (206 nt sequences) (Table S5) were utilized. Both sequence types revealed a grouping into two main clades (I and II) (Figure 2B and Table 7). Clade I comprised sequences from Myanmar, China, and Hungary. In contrast, clade II, the predominant and cosmopolitan clade, included five PPV2 sequences from the present study (OR349215 to OR349219) along with other sequences from Colombia (ON210855 to ON210860), USA (JX101461), Brazil (NC025465), and Republic of Korea (KY018936) (Figure 2B). Clade II was further subdivided into four subclades (I, II, III, and IV). Analysis of the PPV2-VP aa sequences revealed five residues (E712Q, G717A, M784I, S788N, and Q796E) responsible for distinguishing between clade I and clade II.

For PPV3, analysis over 52 NCG and complete sequences (Table S6) yielded an estimated average evolutionary rate of 5.57 × 10^−4^ nspy, with a 95% HPDI. The TMRCA for the phylogenetic evolutionary tree was calculated to be approximately June 1983, based on the most recent analyzed sequence (2021), with a 95% HPDI spanning from December 1971 to December 1991 (Figure 3C). Phylogenetic classification analyses were performed on PPV3-NCG (72 nt sequences) and PPV3-VP (122 nt sequences) (Table S7) for phylogenetic classification, including six novel sequences reported in this study for both analyses. In both cases, PPV3 was divided into two major clades (I and II) (Figure 2C and Table 7). Clade I comprised sequences exclusively from China; whereas, clade II (cosmopolitan) encompassed sequences from diverse geographical origins (Figure 2C). Clade II was further subdivided into five subclades (I, II, III, IV, and V) whose distribution appeared to correlate with distinct geographic regions.

The six Colombian sequences reported in this study were distributed across two subclades of clade II. Three sequences (OR34922, OR34923, OR34926) in subclade I exhibited high identity (99.3%) with sequences from Europe (JF738366) and China (KX827772). The remaining three sequences (OR34924, OR34925, OR34927) were located in subclade V, sharing high identity with sequences from Republic of Korea (OP377042), the USA (KF581754), and Brazil (KY586145). Subclade II predominantly included sequences from Asia, while subclades III and IV clustered sequences from Europe. Analysis of the PPV3-VP aa sequences revealed residues responsible for distinguishing between clades. Notably, five Chinese sequences (JQ177078 to JQ177082) clustered in clade I exhibited two unique residues (L125F and G200D).

Regarding PPV4, analysis of over 32 NCG and complete sequences (Table S8) yielded an estimated evolutionary rate of 3.03 × 10^−4^ nspy. The TMRCA for the phylogenetic evolutionary tree was estimated to be approximately December 1997, based on the most recent analyzed sequence (2021) with a 95% HPDI spanning from August 1983 to November 2004 (Figure 3D). The classification analysis of PPV4-NCG (41 nt sequences) and PPV4-VP (87 nt sequences) (Table S9) revealed a division into three distinct clades (I, II, and III) (Figure 2D and Table 7). Clade I (European) comprised sequences from Hungary, Serbia, Croatia, Poland, and the United Kingdom, while clades II and III were each divided into two subclades (I and II). Both clades encompassed sequences from Asia, Europe, and America. Four novel Colombian sequences of PPV4 are reported in this study. Two of these sequences (OR349293 and OR349294) clustered in clade II, subclade II, exhibiting high identity (99.5%) with sequences from Vietnam (MT434666), Romania (JQ868711), and Hungary (701343). The remaining two sequences (OR349292 and OR349295) were situated in clade III, subclade I, sharing a high identity (99.6%) with sequences from Republic of Korea (OP377033) (Figure 2D). Analysis of the PPV4-VP aa sequences revealed that clade I (European) is characterized by two unique residues (E455Q/D and R200D/R).

For PPV5, analysis of over 42 NCG and complete sequences (Table S10) yielded an estimated evolutionary rate of 2.88 × 10^−4^ nspy. The TMRCA for the phylogenetic evolutionary tree was estimated to be approximately January 1989, based on the most recent analyzed sequence (2021) with a 95% HDP spanning from November 1970 to December 1998 (Figure 3E). The classification of PPV5 was conducted using PPV5-NCG (95 nt sequences) and PPV5-VP (100 nt sequences) (Table S11), resulting in segregation into three distinct clades (I, II, III) (Figure 2E and Table 7). Clades II and III were further subdivided into two subclades (I and II). Interestingly, all three clades harbored strains from diverse continental origins. Among the nine novel Colombian PPV5 sequences reported in this study, one sequence (OR3556159) was located in clade I, exhibiting high identity with sequences from China (MW853953 and MK3782959). Six sequences were grouped in clade II: two (355509 and OR355612) were included in subclade I, showing high identity (99.4 to 99.6%) with sequences from China (MK378344) and the USA (JX896318), while the remaining four were classified in subclade II alongside sequences from China (MK378338) and the USA (MW051673). The final two Colombian strains (OR355610 and OR355614) were assigned to clade III, subclade II, presenting greater identity (99.28%) with sequences from China (MK378336).

Phylogenetic classification analysis of PPV6-NCG (66 nt sequences) and PPV6-VP (130 nt sequences) (Table S13) distinguished two major clades (I and II). Clade I was further divided into two subclades (I and II), while clade II was subdivided into three subclades (I, II, III) (Figure 2F and Table 7). Notably, within clade I, subclade I contained sequences exclusively from China, while subclade II grouped sequences from diverse continental origins. In clade II, subclades I and II harbored cosmopolitan sequences; whereas, subclade III sequences were exclusively of Chinese origin. The three novel Colombian PPV6 sequences reported in this study clustered in clade II. Two of those sequences (OR35618 and OR355619) were assigned to subclade I, exhibiting high identity with sequences from Brazil (KY094494) and the USA (KR709262). The third PPV6 Colombian sequence (OR355617) was placed in subclade II, showing high identity with sequences from Republic of Korea (MH921906) and Poland (KX384819). Analysis based on PPV6-VP aa sequences revealed no specific residues for differentiation between clades. However, examination of PPV6-NCG sequences revealed that most subclade III (clade II) sequences presented a 12 nt deletion (positions 6165 to 6178) in the *3*’UTR region.

For PPV7, analysis of 49 NCG and complete sequences (Table S14) yielded an estimated evolutionary rate of 3.90 × 10^−^^3^ nspy. The TMRCA for the phylogenetic evolutionary tree was estimated to be approximately October 1991, based on the most recent analyzed sequence (2021), with a 95% HDP spanning from May 1985 to October 1995 (Figure 3G). The Bayesian phylogenetic reconstruction for PPV7 encompassed sequences exclusively from China and the two novel Colombian sequences generated in this study. Phylogenetic classification of PPV7-NCG (146 nt sequences) and PPV7-VP (185 nt sequences) (Table S15) resulted in two main clades (I and II), each further subdivided into two subclades (I and II) (Figure 2G and Table 7). In clade I, subclade l grouped sequences only from China, while subclade II grouped sequences from diverse geographical origins.

The two novel Colombian PPV7s (OR349220 and OR349221) reported in this study were assigned to clade I, subclade II, exhibiting high identity with sequences from China (MK092481). Significant variation was observed at the PPV7-VP aa level, with sequences differing in length. The Colombian PPV7-VP aa sequences, OR349221 and OR349221, were 469 and 470 aa in length, respectively. These sequences differed from previously reported Colombian PPV7-VP sequences from 2015 and 2019 (MT758695 and MT758697, respectively), which were 475 aa in length.

Figures S1 and S2 present additional phylogenetic trees based on nPPV-NCG and nPPV-VP (PPV2 through PPV7) with statistical support.

### 2.3. nPPVS Recombination Detection

Analysis of potential recombination events using the pairwise homoplasy index (PHI) test did not provide evidence of recombination in datasets containing the sequences reported in this study. This statistical approach, which evaluates the compatibility of neighboring sites within a sequence alignment, suggests that the novel sequences identified herein have not undergone detectable recombination events.

## 3. Discussion

Phylogenetic investigations into nPPVs have been limited, primarily due to the novelty of the subject, the scarcity of reported sequences, the predominance of partial genomes among these sequences, and the low rate of diagnosis of these viruses [22,29]. Nevertheless, the current study has revealed the endemic circulation of nPPVS within Colombian pig herds, with a particular prevalence among gilts. These findings provide contemporary insights into the phylogenetic classification and molecular evolution of the nPPV genome.

### 3.1. nPPVs as Etiologic Agents, Prevalence, and Coinfections

With regard to nPPVs, it is relevant to establish that their detection rate varies among the published research; this is explained by factors, such as the population evaluated (domestic pigs or wild boars), the sample collected, and the diagnostic techniques carried out [25,31,55]. The above dispersion generates the need to unify criteria, particularly in diagnosing nPPVs, to establish their natural association with diseases. Regarding the latter, the relationship between nPPVs and disease causality remains to be elucidated; for now, PPV2 is at the forefront. Since its discovery [9], PPV2 has been proposed as a candidate etiology of PRDC [56]. This hypothesis is supported by several pieces of evidence, such as (i) the detection of high titers of anti-PPV2 Abs in 40- to 50-day-old pigs suffering from PRDC [57], (ii) the significant prevalence of PPV2-DNA in the lungs of pigs suffering from PRDC [13], and (iii) the identification of PPV2-mRNA in the lung tissue from PRDC cases, demonstrating viral replication mainly in alveolar macrophages and lymphocytes [14,15]. In addition to the particularities mentioned for PPV2, preliminary investigations have assessed the effect of nPPVs on porcine productive parameters and their association with the primary PRDC viruses, such as PCV2 and PRRSV [16].

The relationship between the identification of nPPVs and clinical or subclinical signs of PRF has yet to be established. Evidence shows that all nPPVs (PPV2 through PPV7), except PPV8, have been detected in both the fetal tissue [58] and sera of pregnant sows [59]; these findings are the support for postulating their participation in PRF. It is evident that both the information and the evidence of causality are limited, and it is necessary to conduct research under controlled conditions to verify or refute these proposals. However, none of the nPPVs have been isolated at present, which will delay the process of obtaining conclusive results. On the other hand, research has been conducted to establish the participation of nPPVs in concurrence with other viruses and whether this phenomenon could have an impact on PRF, particularly coinfections involving nPPVs and putative SMEDI viruses, such as PCV2, PRRSV, and PPV1, as well as potential ones, such as PCV3 [58,60]. In that sense, both PPV5 and PPV6 have been proposed as candidates, since they have been detected in coinfection with PCV2, PCV3, and PRRSV in both the fetal tissue samples and sera of pregnant sows suffering from SMEDI [32,59]. At the same time, PPV7 has been identified in sows affected by SMEDI and PPV3/PPV7 coinfection [61].

In most herd-based investigations where nPPVs have been identified, the detection was performed on serum, fecal, and oral fluid samples, mainly in cohorts of breeding and finishing pigs [29,62]. In the present study, their detection was performed by PCR on serum samples from gilts looking for viremia, assuming that serum is a significant sample for viral detection. It is essential to clarify that the tropism of nPPVs remains unclear; therefore, the optimal sample remains to be determined. This lack of understanding of the biological behavior of nPPVs becomes another limitation in selecting an adequate sample for their detection, as in the present study by PCR. A notable finding of our study was that only 30% of the sera examined tested all PPV-negatives, highlighting their high prevalence in Colombian herds. Among nPPVs, PPV3 emerged as the most prevalent (40%), followed by PPV5 (20.5%) and PPV6 (17%), both in individual gilts and herds. These findings align with prior studies where PPV3 similarly dominated [29,63]. For instance, one study on finishing pigs reported a 17.5% prevalence of PPV3 [63], while another found PPV3 and PPV6 to be the most prevalent by up to 10.5% [29]. Comparatively, our study revealed a higher percentage of PPV3-positive samples (40%), potentially attributed to the specific population targeted (gilts aged 180–210 days) compared to the sows [29] or finishing pigs [63] of the two studies mentioned. Our results highlight the potential role of gilts in PPV3 epidemiology.

Another noteworthy aspect is that monoinfections involving nPPVs were detected among gilts in 41%, predominating PPV3 and PPV5; whereas, coinfections involving only PPVs ranging from dual to quadruple, with PPV3 being the most prevalent, followed by PPV5, PPV1, PPV2, and PPV4. As already stated, the impact of viral coinfections involving different PPVs remains understudied and warrants further investigation. However, we insist that these could differentially influence disease patterns and affect the modulation of immune responses compared to PPV monoinfections [64]. In addition to the above, another striking finding in the current study was that the majority of nPPV-positive gilts in mono- and coinfections did not manifest clinical signs of disease, such as PRF or PRDC; that is, they were clinically asymptomatic or with subclinical infection. In this regard, two hypotheses may be proposed: one suggests that nPPVs lack pathogenic effect, serving as commensal viruses as members of the porcine virome [37,65]; the other suggests that these viruses induce persistent infections [66]. Of course, it cannot be ruled out that they are involved as disease etiologies, contributing to conditions such as PRF [58] or PRDC [56,67] with a range of possibilities: in nPPV monoinfections, in nPPV coinfections of PPVs, in nPPV coinfections involving putative pathogens (PCV2, PPV1, and PRRSV) and with diverse clinical signs according to each of these possibilities.

### 3.2. Phylogeny and Evolution of nPPVs

Phylogenetic studies of nPPVS are limited. However, existing research demonstrates the rapid evolution and adaptation of these viruses within swine populations [11,68]. This phenomenon may be attributed to various factors, including high substitution rates comparable to those observed in single-stranded RNA viruses [11,68], positive selection pressures [69], and recombination events [18]. Regarding substitution rates, previous studies have reported nspy of 10^−^^4^ [11,68] for PPV2 through PPV6, while PPV7 exhibited a higher nspy of 10^−^^3^ [68,70], the highest among the nPPVs. Our study corroborates these findings, as comparisons between Colombian PPV7 strains and those cataloged in the GenBank revealed sequence identities ranging from 93.5 to 99.8, the broadest range among all viruses examined in this study. The precise mechanisms driving mutations in nPPVs remain to be elucidated, but they may arise from antigenic escape or a host adaptation process. These mutations can potentially alter viral antigenicity, conferring an evolutionary advantage [11]. Phylogenetic analyses focusing on the VP region of nPPVs have indicated that this region in PPV2, PPV4, and PPV7 is subjected to a strong purifying selection, alongside the presence of positively selected sites [11,70].

In our investigation into the evolution and phylogeny of nPPVs, we observed distinct clustering patterns for all viruses examined. Regarding PPV2, its evolutionary origin is estimated to date back to 1984; although, this remains to be formally documented. Phylogenetically, we propose a PPV2 classification into two distinct clades (I and II), diverging from the previous proposal of seven clades [17], but aligning with the delineation into two clades (A and B), suggested by prior studies [18,19]. These studies emphasized the crucial role of specific residues within the VP region in distinguishing between PPV2 clades. The aforementioned studies [18,19] identified three residues at positions M784I, S788N, and Q796E within the PPV2-VP region. In addition to these findings, our study proposes two additional residues at positions E712G and G717A. These observations lend support to the hypothesis that the PPV2-VP region serves as a potential genotype subtyping region, corroborating previous suggestions [25]. Regarding PPV3, its emergence was estimated to be in 1983. Our study identified significant variability in the NS region, prompting its consideration as a key reference for phylogenetic classification [26]. However, an expanded PPV3-NCG and VP sequences analysis revealed improved classification efficacy based on the aa variability within the VP region. This led to a PPV3 division into two distinct clades (I and II), contrasting with previous proposals of four (A to D) [18] or three groups (1 to 3) [25]. Clade I comprised sequences exclusively from China, characterized by specific aa residues within the VP gene. In contrast, clade II exhibited a cosmopolitan distribution, further subdivided into five subclades based on geographic regions. An evolutionary origin for PPV4 was estimated around 1997. Earlier studies reported minimal nt variations, attributing this to the virus’ adaptation to pigs [22]. However, contrasting views suggest that despite low substitution rates, the virus undergoes significant molecular evolutionary changes [18]. Our study, while confirming PPV4’s relatively low substitution rates among the nPPVs, proposes a PPV4 classification into three well-defined clades (I to III), with clade I specific to sequences from Europe, characterized by distinct residues within the VP region (E455Q/D and R200D/R). This classification differs from the previously proposed two groups (1 and 2) [18,25,71]. Regarding PPV5, phylogenetic studies are notably limited. Initial investigations estimated its emergence to be around 1989. Previous studies proposed two clades (A and B) based on the analysis of a small subset of PPV5-VP sequences [36]. Building upon this, our study, utilizing a larger dataset (95 PPV5-NCG and 102 PPV5-VP sequences), proposes three well-defined PPV5 clades (I to III), each comprising sequences from different continents, albeit without evident geographical clustering. For PPV6, its emergence was estimated to be in 1968, rendering it the oldest among nPPVs. Previously, a phylogenetic classification into two clades (A cosmopolitan and B sequences from China) was proposed based on the study of 48 sequences. This study suggested specific aa residues in the VP region and a 12 nt deletion in the 3’UTR region to differentiate between the clades. Our study also identifies two PPV6 clades (I and II), but with the distinction that sequences from different continents are grouped within each clade rather than separated by geographical location. Similar to the previous study, a 12 nt deletion was observed in the 3’UTR region; although, it was only present in sequences from China within clade II and subclade III. It has been suggested that mutations in the 3’UTR region may impact VP-mRNA translation, as observed in the human B19 virus [72]. This suggests that mutations in the 3’UTR region of PPV6 could affect capsid protein synthesis. Regarding PPV7, its emergence was estimated to be in 1991. Previous studies proposed a classification into two clades (1 and 2) [42] and later into six clades (a to f) [45]. More recently, a new classification based on the insertion of 15 nt (resulting in the addition of five aa) within the Cap protein was proposed [44]. However, our analysis of a larger number of sequences (219 PPV7-VP) led us to propose a PPV7 classification into two clades (I and II), which differs from the previous classification based on the presence or absence of the insertion. As mentioned earlier, PPV7 exhibited the highest substitution rates, reflected in the length diversity of PPV7-VP nt sequences, with some sequences incorporating 3, 12, or 15 nt (resulting in one, four, or five aa) at positions 541 to 556 of PPV7-VP. It is important to note that the insertion of these aa in the VP region does not alter its 3D structure [44].

As previously discussed, multiple PPVs can concurrently infect swine populations, a phenomenon documented in both domestic pigs [32,49,73] and wild boars [18,50,55]. This coinfection suggests the potential for recombination events among PPVs, both intra- and interspecies [11,50]. Although studies are limited, recombination events have been reported for PPV2, PPV3, and PPV7 [18,49]. In our study, despite detecting gilts with quadruple PPVs infections, we found no evidence of recombination among the viral sequences of different PPV species identified in Colombia. This observation suggests that such evolutionary events may not yet have occurred in the evaluated herds. This finding can be attributed to the management practices in most Colombian pig herds, which typically operate as closed-cycle systems, where all stages of production occur on the same farm.

The origins of nPPVs and the mechanism by which these viruses have been introduced to specific geographic regions remain elusive [11]. Phyloevolutionary studies have indicated that nPPVs have been circulating for decades, with some strains dating back up to 50 years [18], despite their recent emergence in various regions worldwide. In Colombia, the presence of nPPVs has been documented since the early 21st century, suggesting a similar evolutionary trajectory to that observed elsewhere, albeit relatively recent within the country’s swine population. This recent emergence may contribute to the limited occurrence of evolutionary and recombination events. Comparative analyses of Colombian nPPVs with those from other continents, such as North America, Europe, and Asia, suggest diverse origins and different introduction times. In the context of pig populations, it is recognized that international trade in live animals, meat, or fomites can facilitate the entry of pathogens into countries [74].

Additionally, it is important to note certain peculiarities within the nPPV species. For instance, PPV4 possesses a circular DNA genome, unlike other PPVs with linear DNA. This attribute may enhance its persistence in pigs [27], as it can persist as episomes in the nucleus of infected cells for extended periods. Furthermore, PPV7 belongs to a distinct viral subfamily compared to other parvoviruses identified thus far, due to its unique characteristics within the *Parvoviridae* family [1].

### 3.3. PPV1

The vaccination of breeding sows using inactivated vaccines and gilt acclimation through exposure to feedback (manure, fetuses, and other tissue) has proven to be the most effective strategy for controlling PPV1 [4]. However, the current study provides evidence of PPV1–viremia in replacement gilts in 17% of serum samples. These results contradict previous studies that have reported a low prevalence of PPV1 attributed to routine herd vaccination programs [65] and effective maternal-derived Abs transfer [75,76]. These Abs are predominantly present in colostrum [77] and exhibit a gradual decline with the piglets’ age, being detectable up to 12 weeks [78]; although, some studies report Abs persistence up to 21 weeks [79]. The impact of PPV1 on FRP is directly related to both the pregnancy stage, where the infection occurs, and the virulence of the viral strain [77,80,81,82,83]. Our findings suggest that, despite mass vaccination programs in Colombia (immunization of gilts before insemination), PPV1 eradication in pig herds has not been achieved. Our prevalence confirms the complexity of PPV1 control and raises questions about the efficacy of current vaccines. The literature indicates that since the early 2000s, severe SMEDI outbreaks have been reported in PPV1-vaccinated herds, particularly in Europe, affecting reproductive parameters, such as the percentage of mummies. These outbreaks are associated with strains referred to as “27a-like” [5]. In this context, it is noteworthy to consider some hypotheses, such as that the vaccines against PPV1 have exerted a selective viral pressure [84], as occurred with the 27a-like viral variants, which have three aa substitutions in VP2, compared to the vaccine strains [5]. These structural modifications could alter the virus’ antigenicity and compromise current vaccines’ efficacy [83]. On the other hand, it has been proposed that the new strains may result from the viral adaptation to the immune response induced by the current vaccines, potentially leading to the emergence of “escape mutants” [4]. The present research also reports, for the first time in Colombia, the ongoing evolution of PPV1, as evidenced by identifying “27a-like” strains. In addition to what has been proposed so far, Streck et al. [84] point out that changes in these viruses may be conditioned not only by evolutionary processes intrinsic to the virus but also by non-evolutionary factors, including the implementation of sanitary measures, the use of disinfectants, and the segregation of pigs into different production categories. These additional factors contribute to the complexity of the epidemiological scenario.

There has yet to be a consensus on the classification of PPV1, with various studies proposing different classification schemes. Consequently, PPV1 has been classified into two clusters (G1 and G2) [5], then into between six and eight clusters (A to H) [6], subsequently, into four lineages (1 to 4) [7], and more recently, into four genotypes (PPV1a to d) [8]. The latter study and our current research are the most comprehensive in analyzing sequences from the complete VP region (V1/VP2/VP3). Our phylogenetic analyses propose a new classification of PPV1 into two clades, I and II, with a clustering aa residue at position 586. Under this new classification, clade I encompasses genotypes PPV1a, PPV1c, and PPV1d, comprising reference sequences with a global distribution. In contrast, clade II includes genotype PPV1b, primarily consisting of sequences reported from Europe since the beginning of this decade, including the 27a-like hypervirulent strains. Notably, the Colombian sequences identified in this study are the only representatives of clade II reported from the American continent thus far. This reclassification highlights the dynamic nature of PPV1. In this context, our study revealed a nspy rate of 10^4^ for the VP region of PPV1, similar to prior studies [6,85].

Finally, despite the contributions of this study, it is essential to acknowledge its limitations. First, the sample size and its focus (replacement gilts) may restrict the generalization of the results to broader age groups. Second, the profile of PPVs found in gilts is only sometimes extended to all pigs in a herd. Third, there may be variations between geographic regions that, together with different management practices, may lead to different adaptations of the viruses studied. The results obtained, together with the limitations, fully justify the launching of research aimed at elucidating the pathogenicity and transmission dynamics of nPPVs and PPV1 variants, with the former providing a comprehensive understanding of the biological characteristics and epidemiology of nPPVs.

## 4. Conclusions

In conclusion, this study aimed to make a significant contribution to our understanding of the genetic diversity of PPV1 and nPPVs within the swine population, focusing on the PPVs identified in Colombia in a cohort of gilts from the five regions that lead swine production in the country. In this crucial population within the production cycle, we identified PPVs (PPV1 through PPV7) in serum samples, indicating active viremia. The most frequently identified nPPV was PPV3, both in monoinfection and in concurrence with other PPVs. Other PPVs identified in order of prevalence were PPV5, PPV1, PPV2, and PPV4. This indicates that these viruses are in circulation within Colombian swine herds. This situation may occur in other latitudes, leading us to propose that further research is necessary to establish whether these viruses play a role in swine pathologies, particularly in PRF.

Additionally, we propose a phylogenetic classification framework for PPVs with international applicability. This contribution is essential, since there is a dispersion of criteria on this topic. In our case, we propose categorizing each type of PPV (PPV1 through PPV7) supported by NCG and VP sequencing.

The findings presented in this study underscore the importance of regularly analyzing and updating the evolutionary changes observed in nPPVs. It is crucial to delineate the genomic alterations and their implications on viral proteins, particularly surface proteins (Capsid), as these modifications can impact virus tropism, interaction with immune cells, and even virulence, as demonstrated for PPV1.

## 5. Materials and Methods

### 5.1. Samples Selection

Serum samples were collected from 234 gilts aged 180 to 200 days across 40 herds in Colombia’s five major swine production provinces (Figure 1). Farms with more than 70 gilts, as determined by the national sow population census (https://www.ica.gov.co/areas/pecuaria/servicios/epidemiologia-veterinaria/censos-2016/censo-2018.aspx) (accessed on 25 April 2023) were designated as primary sampling units and stratified into five categories based on their geographical location (province). Following the random selection of herds, gilts within each herd were considered secondary sampling units. Eight herds were randomly selected from each province, and five to six serum samples were conveniently collected from each selected herd. The sample size for herds and gilts was calculated based on the number of herds with more than 70 gilts in each province. The five selected provinces and the total number of samples collected per province were as follows: Atlántico (*n =* 45), Antioquia (*n* = 47), Cundinamarca (*n* = 41), Eje Cafetero (*n* = 52), and Valle del Cauca (*n* = 49). Serum samples were obtained by centrifugation of blood at 3500 rpm for 10 min and stored at −80 °C until further processing. All evaluated herds were routinely vaccinated against PPV1 at approximately 170 days of age. The number of replacement gilts examined per herd was determined to be six to estimate the presence or absence of the viruses under evaluation. This estimate assumed a between-herd prevalence of 66%, a within herd prevalence of 40%, a test sensitivity of 96%, a test specificity of 99%, and an error of 5%. Sample size calculations were conducted using a hypergeometric distribution and implemented using the “epiR” package in the R statistical analysis software (version 0.9-93) [86].

### 5.2. Detection of PPV1 and nPPVs

Nucleic acids were extracted from 200 µL of serum using the High Pure Viral Nucleic Acid kit^®^ (Roche, Mannheim, Germany) in accordance with the manufacturer’s instructions. All extractions were subsequently stored at −80 °C until further analysis. Individual detection of PPVs (PPV1 through PPV7) was performed via end-point PCR employing previously reported specific primers described in Table 8; for the detection of PPV8, a nested PCR with specific primers was employed [46]. PCR reactions were conducted in a total volume of 25 μL, comprising 0.25 μL of Taq polymerase (5 U/µL) (Go Taq flexi-Promega, Madison, WI, USA), 5× Taq buffer (2.5 μL), 1 µL of each primer (20 μmol/µL), and 2 μL of extracted DNA at a concentration of 200 ng. Thermal cycling conditions for each PCR consisted of an initial denaturation step at 94 °C for 5 min, followed by 35 cycles of denaturation at 94 °C for 30 s, annealing according to the temperatures described in Table 8 for 30 s, and extension at 72 °C for 30 s. A final elongation step was performed at 72 °C for 10 min.

### 5.3. Sequencing of PPV1 and nPPVs

Complete nt sequencing of the PPV1-VP coding region was performed using specific primers, as previously described [6]. For nPPVs (PPV2 through PPV7), near-complete genome (NCG) sequencing was conducted utilizing specific primers previously reported for PPV4 [88], PPV5 [89], and PPV7 [42]. Primers for PPV2, PPV3, and PPV6 were designed using the Primer 3 input program [90]. As PPV8 was not detected in the evaluated gilt population evaluated, it was not sequenced. A comprehensive list of primers utilized in this study is provided in Table S16. The genomic amplification strategy for various nPPVs involved partial overlap of different primer pairs, encompassing the most crucial (coding) regions of the viral genomes. This approach yielded genomic sequences with over 92% coverage (Table S17). Complete coverage (100%) was unattainable due to the absence of the two inverted terminal repeats (ITRs). PCR assays for PPV sequencing were conducted in a total volume of 25 µL containing 0.2 µL of Q5^®^ High-Fidelity DNA Polymerase (New England Biolabs; Ipswich, MA, USA) (5 U/μL), 1× Buffer (2.5 μL), 1 µL of each primer (20 μmol/μL), and 2 μL of extracted DNA (~100 ng). PCR conditions comprised initial denaturation at 94 °C for 1 min, followed by 35 cycles of denaturation at 94 °C for 30 s, annealing at 57 °C for 30 s, and extension at 68 °C for 1 min. Post-amplification, PCR products were purified using the QIAquick PCR purification kit^®^ (Qiagen), according to the manufacturer’s instructions. Subsequently, bidirectional Sanger sequencing was performed (SSiGMol-Molecular Sequencing and Analysis Service of Institute of Genetics, National University of Colombia, Bogotá Campus).

### 5.4. Quality Assurance (QA) Protocols

To ensure that no external material was introduced during DNA extraction and PCR procedures, strict quality assurance (QA) protocols were followed throughout the study. All equipment and work surfaces were thoroughly cleaned with DNA-degrading agents before use. Dedicated reagents and pipettes were employed for DNA extraction and PCR setup, minimizing the risk of cross-contamination. Gilt samples from each property were processed separately to prevent further contamination between batches. Once amplified, PCR products were sent directly for sequencing to avoid cross-contamination. DNA extraction, PCR amplification, and post-amplification product handling were performed in physically separated rooms. UV-light-equipped workstations were also used for mix preparation, along with exclusive pipettes dedicated to this process. The sequencing data validated the results obtained after amplification, confirming the integrity of the procedures.

### 5.5. Phylogenetic Analysis of PPV1 and nPPVs

The nt sequences of PPV1 through PPV7 obtained in this study underwent comprehensive analysis. For PPV1, the analysis involved complete PPV1-VP sequences (*n* = 2); these were compared to 94 available sequences for phylogenetic analyses. For the nPPVs (PPV2 through PPV7), analyses included NCG, nPPV-VP, and nPPV-NS sequences. The analyzed sequences (*n* = 31) were deposited in the GenBank database under the following accession numbers: PP921711 and PP921712 (PPV1, *n* = 2); OR349215 to OR349219 (PPV2, *n* = 5); OR349222 to OR349227 (PPV3, *n* = 6); OR349292 to OR349295 (PPV4, *n* = 4); OR355608 to OR355616 (PPV5, *n* = 9); OR355617 to OR355619 (PPV6, *n* = 3); and OR349220 and OR349221 (PPV7, *n* = 2) (Table S18). For nPPVs (PPV2 through PPV7), comparisons were made with 565 nPPV-NCG, 846 nPPV-VP, and 667 nPPV-NS sequences. These sequences were retrieved from the NCBI GenBank nt database and meticulously selected via Nucleotide BLAST (Basic Local Alignment Search Tool) (Tables S2, S5, S7, S9, S11, S13, and S15). Subsequently, the sequences were aligned using MAFFT alignment tools with default settings [91]. The optimal nt substitution model was determined using the GTR (general time-reversible) model, facilitated by the IQ-Tree phylogenetic reconstruction software (v2.3.6) [92]. Phylogenetic reconstruction was performed using a maximum likelihood (ML) method with 1000 ultrafast bootstrap replicates to ensure the robustness and reliability of inferred relationships among the investigated sequences.

### 5.6. Bayesian Analyses of PPV1 through PPV7

To construct a time-scaled phylogeny encompassing all nPPVs (PPV1 through PPV7) identified in this study, the ML trees of NCG were analyzed using TempEst v1.5.3 [93]. This analysis aimed to assess the temporal signal through regression analysis of root-to-tip genetic distance against sampling dates. Outlier sequences were systematically excluded from all datasets. Detailed information for each dataset is provided in Supplementary Tables S3, S4, S6, S8, S10, S12 and S14 and Figures S3–S9. The phylogeny was reconstructed using Bayesian inference with Markov chain Monte Carlo (MCMC) sampling, implemented in the BEAST v1.10.4 package [94]. Model selection analysis was performed using path-sampling (PS) and steppingstone (SS) procedures to identify the most appropriate molecular clock model for the Bayesian phylogenetic analysis. This involved running 100 path steps, each with 1 million interactions [95]. For this analysis, both the strict molecular clock model and the more flexible uncorrelated log-normal relaxed molecular clock model were considered, along with two nonparametric population growth models: (i) the Bayesian skygrid coalescent model and (ii) the standard Bayesian skyline plot (BSP; with 10 groups) [96,97,98]. PS and SS estimators indicated that the Bayesian skyline with an uncorrelated log-normal relaxed molecular clock was the best-fitted model for the dataset under analysis. Subsequently, triplicate MCMC runs of 50 million states each were conducted, with sampling occurring every 5000 steps to ensure stationary and adequate effective sample size (ESS) for all statistical parameters. The results of the three independent runs were combined using LogCombiner v1.10.4, and MCMC chain convergence was assessed using Tracer v1.7.2 [99]. The maximum clade credibility (MCC) trees from the MCMC samples were summarized using TreeAnnotator V1.10.4, with 10% burn-in removal. Finally, the resulting MCMC phylogenetic tree was visualized using the ggtree R package [100,101].

### 5.7. Recombination Analysis in nPPVs

To detect potential recombination events in the genomes of the nPPVs identified in this study, we employed the methodology implemented in Splits Tree software v4.14.6 [102]. Splits Tree 4 utilizes the pairwise homoplasy index (PHI), where a PHI value below 0.05 indicates statistically significant occurrence of recombination [103].

## Figures and Tables

**Table 1:** Summary of currently reported porcine parvoviruses (PPVs), indicating the date of initial reporting and the latest proposed classification for each.

PPVs	Reporting Year	Latest Proposed Classification
PPV1	1969 [53]	PPV1a to PPV1d [8]
PPV2	2001 [9]	Clades A and B [18,19]
PPV3	2008 [20]	Group 1, 2, and 3 [25]
PPV4	2010 [27]	Group 1 and 2 [11,19,25]
PPV5	2013 [33]	Group A and B [36]
PPV6	2014 [37]	Clades I to III [29]
PPV7	2016 [41]	Clades a to f [45]
PPV8	2022 [54]	Not established

**Table 2:** Prevalence by PCR of PPVs (PPV1 through PPV8) in herds and replacement gilts from the five major swine-producing provinces of Colombia.

Virus	% Positive Herds (Positive/All Tested)	% Positive Gilt Sera (Positive/All Tested)
PPV1	17.5 (7/40)	14.5 (34/234)
PPV2	22.5 (9/40)	9.8 (23/234)
PPV3	67.5 (27/40)	40.1 (94/234)
PPV4	15 (6/40)	4.2 (10/234)
PPV5	40 (16/40)	20.5 (48/234)
PPV6	32.5 (13/40)	17 (40/234)
PPV7	5 (2/40)	1.28 (3/234)
PPV8	0 (0/40)	0 (0/234)

**Table 3:** Detection of PPV genomes in mono and coinfections (up to four distinct PPVs) in the serum of gilts from 40 herds in Colombia.

PPVs	PPV1	PPV2	PPV3	PPV4	PPV5	PPV6	PPV7
**PPV1**	17						
**PPV2**	1	10					
**PPV3**	4	7	36				
**PPV4**	0	0	1	1			
**PPV5**	2	1	14	0	22		
**PPV6**	1	0	16	1	1	11	
**PPV7**	0	0	0	0	0	0	0

**Table 4:** Comparison of previously reported phylogenetic classification schemes for PPV1 and the consensus classification proposed in the current study.

	Study from Germany [5]	Study from Romania, Hungary, and Serbia [6]	Study from China [7]	Study from Belgium and Denmark [8]	This Study from Colombia 2024
**Classification**	Cluster I and II	Cluster A to F	Four lineages: 1–4	Four genotypes (PPV1a-PPV1d)	Two clades and two subclades in each clade
**Region of PPV1 analyzed**	VP1 gene (1740 nt)	VP1/VP2 gene (2190 nt)	VP1/VP2 gene (2190)	VP1/2/3 (2190nt)	VP1/2/3 gene (2190 nt)
**Number of sequences analyzed**	15 sequences	50 sequences	75 sequences	170 sequences	96 sequences
**Nucleotide substitution rates**	Unrealized	2.74 × 10^−4^	3.27 × 10^−5^	4.71 × 10^−5^	1.34 × 10^−4^
PPV1-IDT DEU 1964 (AY684872)	Cluster I	Cluster A	Lineage 2	PPV1a	Clade I Subclade II
PPV1-NALD2 USA (NC001718)	Cluster II	Cluster F	Lineage 3	PPV1c	Clade I Subclade I
PPV1 NADL8 USA (ON014603)	Cluster II	Cluster C-E	Lineage 1, 3, and 4	PPV1d	Clade I Subclade II
PPV1 27a like (AY684871)	Cluster II	B	Lineage 1	PPV1b	Clade II Subclade I
PPV1 Colombian (PP921711)	Cluster II	B	Lineage 1	PPV1b	Clade II Subclade II

**Table 5:** Comparison of sequence identities between the NCG, NS, and VP genes of the nPPVs reported in this study and equivalent sequences available in the NCBI GenBank database.

nPPV	Genetic Identity	%NCG * Identity	% NS ^#^ Identity		% VP ^¶^ Identity	
			nt ^§^	aa ^¤^	nt	aa
PPV1	Within Colombian sequences Within worldwide sequences	Unrealized	Unrealized		99.8 96.4–100	100 95.2–100
PPV2	Within Colombian sequences	97.9–99.7	97.8–99.7	97.5–99.7	97.9–99	98–99.7
	Within worldwide sequences	93.5–98.9	93.3–99.1	95.5–98.6	92.5–99.2	94.9–99.1
PPV3	Within Colombian sequences	97.7–99.8	97–99.8	98.1–100	98.1–99.9	99.2–99.9
	Within worldwide sequences	97.3–99.4	96.2–99.2	96–100	97.6–99.4	98.6–100
PPV4	Within Colombian sequences	98.9–99.8	99.2–99.9	99.3–99.8	98.7–99.9	98.7–100
	Within worldwide sequences	96.1–99.6	96.3–99.9	97.2–100	94.5–100	97.9–100
PPV5	Within Colombian sequences	98–99.8	98.9–100	99.7–100	98.9–99.8	97.6–99.4
	Within worldwide sequences	97.8–99.9	97.1–100	97.6–100	97.8–99.8	96.3–99.8
PPV6	Within Colombian sequences	98.7–99.7	99.7–99.9	99.5–99.8	98.5–99.7	99.3–99.9
	Within worldwide sequences	96.6–99.4	98.9–99.9	99.1–99.8	95.9–99.2	96.5–99.3
PPV7	Within Colombian sequences	95.4–97.4	95–98.2	94.3–98.6	92.4–98.2	91.7–98.9
	Within worldwide sequences	93.5–99.8	93.8–99.9	91.6–99.7	90.4–99.6	87.8–99.8

* NCG: near-complete genomes, # NS: nonstructural protein gene, ^¶^ VP: viral protein (structural), ^§^ nt: nucleotides, ^¤^ aa: amino acids.

**Table 6:** Comparison of nucleotide substitution rates and estimated most recent common ancestry of PPVs.

	Period Range of Sequences	Mean Substitution Rate	HPD Substitution Rate	TMRCA (Years)
PPV1	1963–2021	1.34 × 10^−4^	5.30 × 10^−5^–1.08 × 10^−4^	78
PPV2	1996–2021	5.74 × 10^−4^	4.74 × 10^−4^–6.71 × 10^−4^	40
PPV3	1999–2021	5.57 × 10^−4^	3.63 × 10^−4^–6.50 × 10^−4^	41
PPV4	2007–2021	3.03 × 10^−4^	1.11 × 10^−4^–3.73 × 10^−4^	27
PPV5	1999–2021	2.88 × 10^−4^	1.14 × 10^−4^–3.48 × 10^−4^	35
PPV6	1998–2021	4.22 × 10^−4^	2.18 × 10^−4^–5.36 × 10^−4^	56
PPV7	1997–2021	3.90 × 10^−3^	2.81 × 10^−3^–4.97 × 10^−3^	33

**Table 7:** Comparative analysis of previously reported phylogenetic classification schemes for nPPVs (based on publications with at least two PPVs with proposed classification) and the consensus classification proposed in the current study. Classifications based on the sequences of genes encoding the viral protein (VP) of nPPVs.

	Study from Romania (Cadar et al., 2013) [18]	Study from China (Sun et al., 2015) [19]	Study from Europe (Tregaskis et al., 2021) [25]	Study from Korea (Kim et al., 2022) [29]	Study from China (Chen et al., 2023) [36]	Current study from Colombia (2024) Statistical Support Values (≥%)
PPV2	Clusters A and B 2 subclusters in A 2 subclusters in B 15 sequences analyzed	Clusters A and B 2 subclusters in A 2 subclusters in B 12 sequences analyzed	Clades 1 and 2 114 sequences analyzed (*partial variable region)	Clades 1 and 2 20 sequences analyzed	Clusters A and B (15 sequences)	Clades I (≥81) and II (≥70) 4 subclades in clade II (I ≥ 73, II ≥ 70, III ≥ 79, and IV ≥75) 206 sequences analyzed
PPV3	Clusters A, B, C, and D 2 subclusters in B 2 subclusters in C 3 subclusters in D 38 sequences analyzed	Cluster A and B 2 subclusters in B 2 subclusters in C 3 subclusters in D 14 sequences analyzed	Clades 1, 2, and 3 69 sequences	Not divided into clusters 30 sequences analyzed	Clusters A and B (19 sequences)	Clades I (≥74) and II (≥70) 5 subclades in clade II (I ≥ 86, II ≥ 70, III ≥ 76, IV ≥ 73, and V ≥ 71) 122 sequences analyzed
PPV4	Clusters A and B 20 sequences analyzed	Clusters A and B 11 sequences analyzed	Clades 1 and 2 49 sequences	Not divided into clusters 18 sequences analyzed	No data	Clades I (≥78), II (≥78), and III (≥73) 2 subclades in clade II (I ≥ 78 and II ≥ 80) 2 subclades in clade III (I ≥ 73 and II ≥ 82) 87 sequences analyzed
PPV5	No data	No data	No data	Not divided into clusters 17 sequences analyzed	Clusters A and B (8 sequences)	Clades I (≥80), II (≥71), and III (≥72) 2 subclades in clade II (I ≥ 72 and II ≥ 77) 2 subclades in clade III (I ≥ 77 and II ≥ 72) 100 sequences analyzed
PPV6	No data	No data	No data	Clades 1, 2, and 3 27 sequences analyzed	No data	Clades I (≥86) and II (≥72) 2 subclades in clade I (I ≥ 90 and II ≥ 86) 3 subclades in clade II (I ≥ 72, II ≥ 73, and III ≥ 72) 130 sequences analyzed
PPV7	No data	No data	No data	Clades 1, 2, and 3 21 sequences analyzed		Clades I (≥72) and II (≥70) 2 subclades in clade I (I ≥ 65 and II ≥ 72) 2 subclades in clade II (I ≥ 71 and II ≥ 70) 185 sequences analyzed

**Table 8:** List of specific primers used in the current study for the detection of porcine parvoviruses (PPV1 through PPV8).

Virus	Primer Name	Sequence	Annealing Temp	Bp Size	Reference
PPV1	PPV1F	CTTGGAGCCGTGGAGCGAGC	60	147	[87]
PPV1	PPV1R	TGCACAGTTTTCACCAAAGCAGGC			
PPV2	PPV-2-F	AGCTCTGCGACAAGTGGG	58	563	[56]
PPV2	PPV-2-R	GTCTACGGCCTGCAAGAA			
PPV3	PPV-3-F	CCACGCCAAATCAAAGTC	58	514	
PPV3	PPV-3-R	CTCCCACTCCCATCCACT			
PPV4	PPV-4-F	TATGTGGGCTGGGCAAGGAATGTC	58	416	
PPV4	PPV-4-R	GTTGCGGAATGCTATCAGGCTCTT			
PPV5	PPV-5-F	ACACCTCCTGCGGCTTAT	58	959	
PPV5	PPV-5-R	GTGTAGCGATGTCCTGGC			
PPV6	PPV-6-F	CTTTGGTGTAGAGGGCTTGA	58	650	
PPV6	PPV-6-R	CGGTTGTAGCAGGTCCAA			
PPV7	PPV7F	CCTCCATCAGCAGCGACCAGT	58	241	[42]
PPV7	PPV7R	ACCAGGGTTCCGTTTTCGTCT			
PPV8	PPV8-outF	TGTTGGTTTGCACCTAGCG	58		[46]
PPV8	PPV8-outR	TGATGAGATGGTGGAACGC			
PPV8	PPV8-inF	TCCAAGTTGCCCTAGACAGC	58	554	
PPV8	PPV8-inR	GCCTCGTACATGTGGACCTC			

## Data Availability

The original contributions presented in the study are included in the article/Supplementary Materials. Further inquiries can be directed to the corresponding author.

## Supplementary Materials

The following supporting information can be downloaded at: https://www.mdpi.com/article/10.3390/ijms251910354/s1.
