# Supplementary material for: Molecular Epidemiology and Phyloevolutionary Analysis of Porcine Parvoviruses (PPV1 through PPV7) Detected in Replacement Gilts from Colombia
Source: Int J Mol Sci. 2024 Sep 26;25(19):10354. doi: 10.3390/ijms251910354 (PMC11476972; doi:10.3390/ijms251910354)
Supplement: Supplementary file 1 [file ijms-25-10354-s001.zip › ijms-3182044-supplementary.pdf]

Table S1. Detection of triple and quadruple coinfections involving PPVs found in the serum (n = 234) of replacement gilts from five major swine-producing provinces (40 herds) in Colombia.

Infection status	Number of sera	Viral coinfection (number of sera)
Triple infection	15	PPV1/PPV3/PPV6 (4), PPV2/PPV3/PPV5 (3), PPV1/PPV3/PPV4 (2), PPV1/PPV3/PPV5(2), PPV2/PPV3/PPV4 (1), PPV3/PPV5/PPV6 (1), PPV4/PPV5/PPV6(1), PPV4/PPV6/PPV7 (1),
Quadruple infection	3	PPV1/PPV3/PPV6/PPV7 (1), PPV3/PPV4/PPV5/PPV6 (1), PPV3/PPV4/PPV6/PPV7 (1)

Table S2. List and identification of 94 PPV1-VP complete gene sequences sampled from NCBI – GenBank database and compared by phylogenetic analysis with two PPV1-VP complete sequences from Colombia (recovered from the current study).

Accession	Year	Country
AY684864.1	2002	Germany
AY684867.1	2002	Germany
AY684869.1	2002	Germany
AY684870.1	2002	Germany
AY684871.1	2001	Germany
AY684872.1	1964	Germany
GQ884035.1	2005	Europe
JN400520.1	2009	Germany
JQ249921.1	2011	Romania
ON014668	2016	Denmark
ON014670	2006	Denmark
ON014667	2015	Denmark
ON014672	2009	Denmark
ON014666	2021	Denmark
ON014686	1976	Denmark
ON014660	1983	Denmark
ON014679	2021	Denmark
ON014615	1972	Germany
ON014618	NA	NA
ON014682	2021	France
AY684865.1	2005	Germany
AY684868.1	2006	Germany
GQ884036.1	2005	Europe
GQ884041.1	2006	Europe
GQ884045.1	2006	Europe
HM627652.1	2010	China
JN400516.1	2009	Germany
JN400518.1	2009	Germany
JQ249915.1	2011	Romania
JQ249927.1	2011	Romania
KC296744.1	2010	Germany
KC296746.1	2010	Germany
ON014641	2017	Denmark
ON014630	2019	Denmark
ON014629	2019	Denmark
ON014602	2020	Denmark
ON014651	2017	Denmark
ON014683	2017	Denmark
ON014642	2018	Denmark
ON014638	2020	Denmark
ON014631	2016	Denmark
ON014675	2021	Denmark
ON014674	2021	Denmark
ON014604	2020	France
ON014606	2020	The Netherlands
ON014613	2020	Germany
ON014611	2021	The Netherlands
ON014608	2021	France
ON014610	2021	Belgium
ON014612	2019	Belgium
ON014614	2019	The Netherlands
ON014619	2021	The Netherlands
ON014620	2021	France
ON014688	2021	The Netherlands
EU790642.1	2004	China
HM989009.1	1989	China
JN968975.1	2010	China
JX992846.1	2008	China
KF429252.1	2012	China
KF742500.2	2013	China
KJ201927.1	2009	China
KX233726.1	2015	China
KX242359.1	2013	China
KY994646.1	2016	South-Korea
MF447833.1	2013	China
MH566237.1	2018	South-Korea
MK993540.1	2017	China
NC_001718.1	1964	USA
MW711830.1	2017	South-Korea
AY390557.1	2003	South-Korea
AY583318.1	2004	China
AY684866.1	2005	UK
DQ675456.1	2006	China
EU790641.1	2006	China

ON014634	2019	Denmark	GQ884042.1	2006	Europe
ON014648	2020	Denmark	GQ884045.1	2006	USA
ON014644	2020	Denmark	HM627653.1	2009	China
ON014643	2020	Denmark	JN860197.1	2012	China
ON014655	2016	Denmark	JN872448.1	2010	China
ON014633	2017	Denmark	JQ249913.1	2011	Romania
KF049426.1	2010	Germany	JQ249914.1	2011	Romania
MW711828.1	2018	South-Korea	JQ249922.1	2011	Romania
U44978.1	1985	USA	JQ249926.1	2011	Romania
ON014659	2015	Denmark			
ON014603	1976	USA			

Table S3. List and identification of 114 complete VP Porcine parvovirus 1 (PPV1) genome sequences sampled from NCBI – GenBank database and used in the Bayesian phylogenetic reconstruction.

Accession	Year	Country	Accession	Year	Country
ON014617.1	1963	United Kingdom	MN326127.1	2017	China
ON014603.1	1976	USA	MN326129.1	2017	China
ON014687.1	1976	Denmark	MZ577026.1	2017	China
MN326126.1	1999	China	MT846929.1	2017	South Korea
EU790642.1	2004	China	MT846931.1	2017	South Korea
GQ884045.1	2006	USA	MT846932.1	2017	South Korea
GQ884046.1	2006	USA	MW711828.1	2018	South Korea
GQ884047.1	2006	USA	MN970191.1	2018	Brazil
EU790641.1	2006	China	MH817778.1	2018	South Korea
JX992846.1	2008	China	MH566237.1	2018	South Korea
ON014622.1	2009	Denmark	MZ491178.1	2019	South Korea
HM627653.1	2009	China	MZ491179.1	2019	South Korea
ON014684.1	2010	USA	ON014607.1	2019	Netherlands
JN872448.1	2010	China	ON014612.1	2019	Belgium
JQ249913.1	2011	Romania	ON014624.1	2019	Denmark
JQ249915.1	2011	Romania	ON014627.1	2019	Denmark
JQ249916.1	2011	Romania	ON014629.1	2019	Denmark
JQ249917.1	2011	Romania	ON014630.1	2019	Denmark
JQ249918.1	2011	Romania	ON014634.1	2019	Denmark
JQ249919.1	2011	Romania	ON014652.1	2019	Denmark
JQ249920.1	2011	Romania	ON014602.1	2020	Denmark
JQ249922.1	2011	Romania	ON014604.1	2020	France
JQ249925.1	2011	Romania	ON014605.1	2020	France
JQ249926.1	2011	Romania	ON014606.1	2020	Netherlands
JQ249927.1	2011	Romania	ON014613.1	2020	Germany
KF429253.1	2012	China	ON014632.1	2020	Denmark
MF447833.1	2013	China	ON014635.1	2020	Denmark
MN326132.1	2014	China	ON014636.1	2020	Denmark
MH183294.1	2014	China	ON014638.1	2020	Denmark
MH183296.1	2014	China	ON014643.1	2020	Denmark
MH183297.1	2014	China	ON014644.1	2020	Denmark
ON014623.1	2015	Denmark	ON014648.1	2020	Denmark
ON014628.1	2015	Denmark	ON014653.1	2020	Denmark
ON014640.1	2015	Denmark	MZ577027.1	2020	China
ON014658.1	2015	Denmark	PP316166.1	2021	Viet Nam

Accession	Year	Country
ON014659.1	2015	Denmark
MN326128.1	2015	China
MN326131.1	2015	China
MK092380.1	2015	China
MK092381.1	2015	China
MK092383.1	2015	China
MH183295.1	2015	China
MH183298.1	2015	China
OP377055.1	2016	South Korea
ON982164.1	2016	South Korea
ON014626.1	2016	Denmark
ON014631.1	2016	Denmark
ON014637.1	2016	Denmark
ON014646.1	2016	Denmark
ON014647.1	2016	Denmark
MN326130.1	2016	China
ON014625.1	2017	Denmark
ON014633.1	2017	Denmark
ON014641.1	2017	Denmark
ON014649.1	2017	Denmark
ON014650.1	2017	Denmark
ON014657.1	2017	Denmark

Accession	Year	Country
OR452190.1	2021	China
OR452191.1	2021	China
ON924737.1	2021	China
ON924738.1	2021	China
ON924739.1	2021	China
MZ677266.1	2021	China
MZ706996.2	2021	China
ON014608.1	2021	France
ON014611.1	2021	Netherlands
ON014616.1	2021	France
ON014619.1	2021	Netherlands
ON014620.1	2021	France
ON014621.1	2021	France
ON014639.1	2021	Denmark
ON014673.1	2021	Denmark
ON014674.1	2021	Denmark
ON014675.1	2021	Denmark
ON014676.1	2021	Denmark
ON014681.1	2021	Denmark
ON014683.1	2021	Denmark
PP921711	2021	Colombia
PP921712	2021	Colombia

Table S4. Details of the 74 complete and near-complete genome (NCG) sequences of porcine parvovirus 2 (PPV2) sampled from NCBI and used in the Bayesian phylogenetic reconstruction.

Accession	Year	Country
MN326164	1996	China
MN326183	1996	China
MN326146	1997	China
MN326143	1998	China
MN326148	1998	China
MN326149	1998	China
MN326151	1998	China
MN326156	1998	China
MN326166	1998	China
MN326173	1998	China
MN326175	1998	China
MN326133	1999	China
MN326136	1999	China
MN326137	1999	China
MN326139	1999	China
MN326140	1999	China
MN326145	1999	China
MN326147	1999	China
MN326162	1999	China
MN326165	1999	China
MN326168	1999	China
MN326171	1999	China
MN326174	1999	China
MN326181	1999	China
GU938299	2009	China
JX101461	2011	USA
JX101462	2011	USA
NC_025965	2011	Brazil
KU745627	2015	China
MK092389	2015	China
MK092390	2015	China
MK092391	2015	China
MK092392	2015	China
MK092393	2015	China

Accession	Year	Country
MK092397	2015	China
MK092398	2015	China
MK092399	2015	China
MN326155	2015	China
MN326170	2015	China
KY018935	2016	South Korea
KY018936	2016	South Korea
OP377053	2016	South Korea
MK378157	2017	China
MK378158	2017	China
MK378218	2017	China
MK378224	2017	China
MN326135	2017	China
MW853946	2017	China
MW853948	2017	China
MW853949	2017	China
MH778962	2018	South Korea
MH921914	2018	South Korea
MW051675	2018	USA
MW853944	2018	China
MW883398	2020	USA
MW883399	2020	USA
MZ577030	2020	China
ON210858	2020	Colombia
ON210859	2020	Colombia
ON210855	2021	Colombia
ON210856	2021	Colombia
ON210857	2021	Colombia
ON210860	2021	Colombia
ON210861	2021	Colombia
ON698675	2021	China
ON698677	2021	China
OR349215	2021	Colombia
OR349216	2021	Colombia

Accession	Year	Country
MK092394	2015	China
MK092395	2015	China
MK092396	2015	China

Accession	Year	Country
OR349217	2021	Colombia
OR349218	2021	Colombia
OR349219	2021	Colombia

Table S5. List and identification of 206 PPV2-VP complete gene sequences sampled from NCBI – GenBank database and compared by phylogenetic analysis with five PPV2-VP complete sequences from Colombia (recovered from the current study).

Accession	Year	Country
NC_025965	2011	Brazil
**JX101462**	2011	USA
**JX101461**	2011	USA
**MG345014**	2018	China
**MW051675**	2018	USA
**ON210858**	2020	Colombia
**MW883399**	2020	USA
**MW883398**	2020	USA
**GU938299**	2009	China
**KY018935**	2016	South Korea
**KY018936**	2016	South Korea
**ON210857**	2021	Colombia
**ON210855**	2021	Colombia
**OP377053**	2016	South Korea
**ON210856**	2021	Colombia
**ON210859**	2020	Colombia
**MK378219**	2017	China
**MZ577030**	2020	China
**ON210860**	2021	Colombia
**MW853948**	2017	China
**MH778962**	2018	South Korea
**MW853946**	2017	China
**MW853944.**	2017	China
**MK378158**	2017	China
**ON210861**	2021	Colombia
**MG345018**	2018	China
**MG345013**	2018	China
**MK378224**	2017	China
**KU745627**	2015	China
**MH921914)**	2018	South Korea
**MK378157**	2017	China
**MG345019**	2018	China
**MG345015**	2018	China
**MK378188**	2017	China
**MN326149**	1998	China
**MN326160.1**	1999	China
**MN326143**	1998	China
**MN326137**	1999	China
**ON698675**	2021	China
**MN326181**	1999	China
**MN326175**	1998	China
**MN326147**	1999	China
**MK378218**	2017	China
**MN326183**	1996	China
**MN326151**	1998	China
**MN326133**	1999	China
**ON698677**	2021	China
**MN326146**	1997	China
**MN326140**	1999	China
**MK092389**	2015	China
**MN326174**	1999	China
**MK092386**	2015	China
**MG345017**	2017	China
**MN326171**	1999	China
**MN326145**	1999	China
**MK092395**	2015	China
**MN326164**	1996	China
**MK092390**	2015	China
**MN326176**	2014	China
**MN326139**	1999	China
**MN326136**	1999	China
**MK092391**	2015	China
**MK092393**	2015	China
**MN326148**	1998	China
**MN326165**	1999	China
**MN326153**	1998	China
**MK092397**	2015	China
**MN326168**	1999	China
**MN326166**	1998	China
**MN326172**	1998	China
**MK092398**	2015	China
**MK092399**	2015	China
**MK092396**	2015	China
**MN326156**	1998	China
**MN326173**	1998	China
**AB916464**	2010	Japan
**MK092394**	2015	China

**MN326141**	2017	China	**OP377049**	2016	South Korea
**MN326169**	1999	China	**MK092388**	2015	China
**MN326162**	1999	China	**MW893680**	2020	China
**MK092392**	2015	China	**ON698678**	2021	China
**MN326178**	2015	China	**MK378217**	2017	China
**MK378162**	2017	China	**MN326159**	2017	China
**MZ577028**	2017	China	**MK378187**	2017	China
**MG345016**	2018	China	**MH778963**	2018	South Korea
**MN326170**	2015	China	**MN326180**	2015	China
**MZ577029**	2019	China	**KY586144**	2008	Brazil
**MK378159**	2017	China	**MK378206**	2017	China
**MN326155**	2015	China	**MN326158**	2015	China
**MK378179**	2017	China	**MN326144**	2017	China
**MW853943**	2017	China	**MN326177**	2015	China
**MK378172**	2017	China	**MN326167**	2015	China
**MN326157**	2016	China	**MN326152**	2015	China
**GU938301**	2010	China	**AB076669**	2000	Myanmar
**NC_038883**	2009	China	**MN326142**	2017	China
**KP245947**	2007	China	**MK378189**	2017	China
**MW853947**	2017	China	**MW853949**	2017	China
**MN326135**	2017	China	**MK378213**	2017	China
**MK378163**	2017	China	**KP245942**	2012	China
**MW030464**	2018	China	**MN326191**	1998	China
**MW853945**	2017	China	**MK092407**	2015	China
**MK378207**	2017	China	**MN326184**	2017	China
**OP377050**	2017	South Korea	**MN326190**	1998	China
**MK378208**	2017	China	**JQ860245**	2009	Romania
**MK378197**	2017	China	**KP245945**	2013	China
**ON698674**	2021	China	**MN326189**	2017	China
**MK092401**	2015	China	**MK378160**	2017	China
**MK092400**	2015	China	**OP377046**	2020	South Korea
**MK378220**	2017	China	**MZ491184**	2018	South Korea
**MK092402**	2015	China	**MZ491181**	2018	South Korea
**ON698676**	2021	China	**JQ860244**	2009	Romania
**OP377052**	2016	South Korea	**KC701304**	2011	Serbia
**MK378211**	2017	China	**MN326192**	2015	China
**OP377051**	2016	South Korea	**KC701310**	2007	Hungary
**MN326138**	1999	China	**MK378215**	2017	China
**KX517759**	2013	Hungary	**MN326187**	2017	China
**NC_035180**	2013	Hungary	**KC701307**	2008	Croatia
**KP765690**	2013	Hungary	**MK378168**	2017	China
**MK092387**	2015	China	**MZ491183**	2018	South Korea
**MK092385**	2015	China	**KC687099**	2009	Croatia

**MZ491182**	2018	South Korea	**KC701292**	2007	Serbia
**KC701308**	2011	Croatia	**JQ860247**	2009	Romania
**KC687097**	2011	Poland	**KC701296**	2009	Hungary
**KC701295**	2011	Poland	**KP245946**	2013	China
**MN326188**	2017	China	**MN326185**	2017	China
**KC701311**	2007	Hungary	**KC767891**	2011	Poland
**KC701309**	2011	Poland	**KC701303**	2011	Poland
**KC701306**	2007	Serbia	**KC701294**	2011	Serbia
**KF725662**	1998	USA	**KC701293**	2011	Serbia
**JQ860246**	2009	Romania	**MK092406**	2015	China
**KC701313**	2008	Serbia	**JQ860248**	2009	Romania
**KC701312**	2008	Serbia	**KC701301**	2011	Poland
**MK378183**	2017	China	**KC701300**	2009	Hungary
**MK092408**	2015	China	**KC701291**	2006	Croatia
**KC701314**	2011	Poland	**KC701297**	2008	Serbia
**KC687098**	2011	Croatia	**KC701298**	2007	Hungary
**JQ860241**	2010	Romania	**OR349215**	2021	Colombia
**JQ860242**	2010	Romania	**OR349216**	2021	Colombia
**JQ860243**	2009	Romania	**OR349217**	2021	Colombia
**MK092405**	2015	China	**OR349218**	2021	Colombia
**JQ860240**	2010	Romania	**OR349219**	2021	Colombia
**JQ860238**	2010	Romania			

Table S6. Details of the 52 complete and near-complete genome (NCG) sequences of porcine parvovirus 3 (PPV3) sampled from NCBI and used in the Bayesian phylogenetic reconstruction.

Accession	Year	Country
MN326197	1999	China
MN326199	1999	China
KY586145	2008	Brazil
JQ868699	2010	Romania
JQ868701	2010	Romania
JQ868703	2010	Romania
KC296750	2010	Germany
KC296751	2010	Germany
JQ868702	2010	Romania
JQ868700	2010	Romania
JQ868704	2010	Romania
KX827777	2015	China
KX827776	2015	China
KX827774	2015	China
KX827775	2015	China
KX827773	2015	China
KX827772	2015	China
MK092411	2015	China
MK092409	2015	China
MK092410	2015	China
MK092412	2015	China
OP377040	2016	South Korea
OP377041	2016	South Korea
OP377042	2016	South Korea
OP377043	2016	South Korea
MH884552	2016	Italy

Accession	Year	Country
MW711834	2017	South Korea
MN326196	2017	China
MK378244	2017	China
MK378230	2017	China
MK378231	2017	China
MK378238	2017	China
MK378232	2017	China
MK378239	2017	China
MK378240	2017	China
MK378226	2017	China
MK378233	2017	China
MN326198	2017	China
MK378234	2017	China
MZ577032	2018	China
MW030463	2018	China
MZ577031	2018	China
MW853950	2018	China
MZ577034	2020	China
MZ577033	2020	China
OR046030	2021	China
OR349222	2021	Colombia
OR349223	2021	Colombia
OR349224	2021	Colombia
OR349225	2021	Colombia
OR349226	2021	Colombia
OR349227	2021	Colombia

Table S7. List and identification of 122 PPV3-VP complete gene sequences sampled from NCBI – GenBank database and compared by phylogenetic analysis with six PPV3-VP complete sequences from Colombia (recovered from the current study).

Accession	Year	Country
**KY586145**	2008	Brazil
**MW711835**	2018	South Korea
**NC_022104**	2010	Canada
**KC992732**	2010	Canada
**MT846935**	2017	South Korea
**MW711834**	2017	South Korea
**JQ425259**	2011	USA
**EU200671**	2007	China
**MT846933**	2017	South Korea
**JF738362**	2010	Romania
**MZ577031**	2018	China
**NC_022104**	2011	USA
**MZ577032**	2018	China
**JQ425258**	2011	USA
**NC_038546**	2008	China
**MK378226**	2017	China
**MZ577033**	2020	China
**EU200672**	2007	China
**EU200676**	2008	China
**EU200675**	2008	China
**MK378240**	2017	China
**N990268**	2011	China
**MK378244**	2017	China
**MG345026**	2017	China
**KU167029**	2015	China
**JF738366**	2011	Romania
**OP377042**	2016	South Korea
**FJ982254**	2000	United Kingdom
**OP377040**	2016	South Korea
**JN990267**	2011	China
**JF738364**	2010	Romania
**JF738351**	2007	Romania
**EU200674**	2007	China
**KU167028**	2016	China
**FJ982251**	1996	United Kingdom
**JN990266**	2011	China
**EU200673**	2008	China
**JF738368**	2010	Romania
**MZ577034**	2020	China
**OP377041**	2016	South Korea
**MK378239**	2017	China
**FJ982252**	1996	United Kingdom
**MN326199**	1999	China
**MK092412**	2015	China
**JF738357**	2010	Romania
**JN990269**	2011	China
**JF738367**	2010	Romania
**FJ982247**	2001	United Kingdom
**FJ982248**	2001	United Kingdom
**FJ982246**	2001	United Kingdom
**MW853950**	2017	China
**MK092410**	2015	China
**MK378232**	2017	China
**MK378230**	2017	China
**FJ982255**	2000	United Kingdom
**FJ982253**	1999	United Kingdom
**FJ982250**	1994	United Kingdom
**FJ982249**	1994	United Kingdom
**MK092409**	2015	China
**MK092411**	2015	China
**KX827775**	2015	China
**MN326197**	2015	China
**MN326196**	2017	China
**KX827773**	2015	China
**GQ869540**	2007	Germany
**MN326195**	1998	China
**MN326193**	1997	China
**MN326198**	2017	China
**KX827776**	2015	China
**KX827774**	2015	China

**KX827772**	2015	China
**MN326194**	1998	China
**KF225551**	2008	Germany
**GQ869539**	2005	Germany
**JQ177078**	2011	China
**KX827777**	2015	China
**MK378231**	2017	China
**JQ177079**	2011	China
**MG365914**	2016	China
**KF225548**	2012	Cameroon
**MK378238**	2017	China
**KF225549**	2012	Cameroon
**MG365915**	2016	China
**MK378233**	2017	China
**MG365916**	2017	China
**MG365919**	2016	China
**JQ177080**	2010	China
**MG365917**	2017	China
**MW030463**	2018	China
**MG365920**	2017	China
**KC296752**	2010	Germany
**MH884552**	2016	Italy
**KC296751**	2010	Germany
**KC296750**	2010	Germany
**JQ177081**	2011	China
**JQ177082**	2011	China
**JQ177083**	2010	China
**KC211901**	2012	China
**OP377043**	2016	South Korea
**JQ868700**	2010	Romania
**MK378234**	2017	China
**JQ868702**	2010	Romania
**JQ868699**	2010	Romania
**JF738350**	2006	Romania
**JQ868701**	2010	Romania
**JQ868704**	2010	Romania
**JQ868703**	2010	Romania
**JQ868705**	2010	Romania
**JQ868706**	2010	Romania
**JF738352**	2006	Romania
**KC211905**	2012	China
**KC211902**	2012	China
**KC211897**	2012	China
**KC211898**	2012	China
**KC211896**	2012	China
**KC211904**	2012	China
**OR349222**	2021	Colombia
**OR349223**	2021	Colombia
**OR349224**	2021	Colombia
**OR349225**	2021	Colombia
**OR349226**	2021	Colombia
**OR349227**	2021	Colombia

Table S8. Details of the 32 complete and near-complete genome (NCG) sequences of porcine parvovirus 4 (PPV4) sampled from NCBI and used in the Bayesian phylogenetic reconstruction.

Accession	Year	Country
JQ868713	2007	Romania
JQ868714	2007	Romania
KY586146	2008	Brazil
GU978965	2009	China
GU978964	2009	China
HM031134	2009	China
HM031135	2009	China
GU978968	2009	China
GU978967	2009	China
OP377033	2016	SouthKorea
OP377034	2016	SouthKorea
OP377030	2016	SouthKorea
OP377032	2016	SouthKorea
MK378249	2017	China
MK378268	2017	China
MK378278	2017	China

Accession	Year	Country
MZ577035	2017	China
MH921902	2017	SouthKorea
MK378257	2017	China
MW711836	2017	SouthKorea
MH921910	2018	SouthKorea
MW073110	2018	USA
MH921915	2018	SouthKorea
MH921911	2018	SouthKorea
MT434669	2019	VietNam
MT434667	2019	VietNam
MT434668	2019	VietNam
MZ577036	2020	China
OR349292	2021	Colombia
OR349293	2021	Colombia
OR349294	2021	Colombia
OR349295	2021	Colombia

Table S9. List and identification of 87 PPV4-VP complete gene sequences sampled from NCBI – GenBank database and compared by phylogenetic analysis with four PPV4-VP complete sequences from Colombia (recovered from the current study).

Accession	Year	Country
**OP377033**	2016	South Korea
**GU978965**	2009	China
**GU978967**	2009	China
**MW073110**	2018	USA
**OP377034**	2016	South Korea
**HM031135**	2009	China
**OP377030**	2016	South Korea
**MH921910**	2018	South Korea
**KY586146**	2008	Brazil
**MK378268**	2017	China
**GQ387500**	2006	USA
**NC_014665**	2006	USA
**GU978966**	2009	China
**MG345027**	2017	China
**MH921902**	2017	South Korea
**JQ868713**	2007	Romania
**MH921911**	2018	South Korea
**JQ868714**	2007	Romania
**MH921915**	2018	South Korea
**JQ868716**	2007	Romania
**JQ868715**	2007	Romania
**GU978964**	2009	China
**GU978968**	2009	China
**HM031134**	2009	China
**MZ577035**	2017	China
**MZ577036**	2020	China
**OP377032**	2016	South Korea
**MT434669**	2019	Vietnam
**MT434667**	2019	Vietnam
**MT434668**	2019	Vietnam
**MK378262**	2017	China
**MW711836**	2017	South Korea
**MK378249**	2017	China
**MK378260**	2017	China
**MT877649**	2017	South Korea
**MT877650**	2017	South Korea
**MK378257**	2017	China
**MK378247**	2017	China
**OP377031**	2016	South Korea
**MK092421**	2015	China
**MK378273**	2017	China
**MK378255**	2017	China
**MK378286**	2017	China
**MK378278**	2017	China
**MK378270**	2017	China
**MK378264**	2017	China
**MK378269**	2017	China
**MK378276**	2017	China
**MK378263**	2017	China
**MK092420**	2015	China
**KC701352**	2011	Poland
**MZ491189**	2018	South Korea
**MZ491188**	2018	South Korea
**KC701346**	2008	Hungary
**KC701336**	2011	Poland
**KC701340**	2007	Hungary
**KC701351**	2007	Serbia
**JQ868710**	2010	Romania
**JQ868709**	2010	Romania
**MZ491190**	2018	South Korea
**MK609918**	2002	United Kingdom
**KC701355**	2011	Poland
**KC701343**	2008	Hungary
**KC701341**	2007	Hungary
**JQ868708**	2010	Romania
**KP245953**	2012	China
**KC701348**	2012	China
**KC701347**	2011	Poland
**KC701344**	2008	Croatia
**KC701333**	2011	Poland
**KC701356**	2007	Serbia
**KC701350**	2011	Poland
**KC701334**	2011	Poland
**KC701354**	2007	Serbia
**KC701337**	2011	Poland
**KC701353**	2007	Serbia
**KC701349**	2007	Hungary

**KC701335**	2011	Poland	**KC701342**	2007	Hungary
**JQ868711**	2007	Romania	**OR359292**	2021	Colombia
**KC701345**	2008	Serbia	**OR359293**	2021	Colombia
**KC701338**	2006	Serbia	**OR359294**	2021	Colombia
**KC701339**	2007	Hungary	**OR359295**	2021	Colombia

Table S10. Details of the 42 complete and near-complete genome (NCG) sequences of porcine parvovirus 5 (PPV5) sampled from NCBI and used in the Bayesian phylogenetic reconstruction.

Accession	Year	Country
MN326214.1	1999	China
JX896321.1	2011	USA
NC_023020.1	2011	USA
JX896320.1	2011	USA
JX896322.1	2011	USA
JX896319.1	2011	USA
JX896318.1	2011	USA
MK092425.1	2015	China
MK092426.1	2015	China
MN326219.1	2015	China
MN326216.1	2015	China
MN326218.1	2015	China
MN326213.1	2015	China
MN326217.1	2015	China
KU745628.1	2015	China
OR046032.1	2016	China
MN326212.1	2017	China
MN326210.1	2017	China
MH921905.1	2017	South Korea
MN326209.1	2017	China
MH921908.1	2017	South Korea
MH921904.1	2017	South Korea
MN326211.1	2017	China
MW051674.1	2018	USA
MT671979.1	2018	Brazil
MW853952.1	2018	China
MW853953.1	2018	China
MW711837.1	2018	South Korea
MW711838.1	2018	South Korea
MW711839.1	2018	South Korea
MH921912.1	2018	South Korea
MW853951.1	2018	China
MZ577038.1	2020	China
OR355608	2021	Colombia
OR355609	2021	Colombia
OR355610	2021	Colombia
OR355611	2021	Colombia
OR355612	2021	Colombia
OR355613	2021	Colombia
OR355614	2021	Colombia
OR355615	2021	Colombia
OR355616	2021	Colombia

Table S11. List and identification of 100 PPV5-VP complete gene sequences sampled from NCBI – GenBank database and compared by phylogenetic analysis with nine PPV5-VP complete sequences from Colombia (recovered from the current study).

Accession	Year	Country
MW051673.1	2019	USA
MZ577037.1	2017	China
JX896319.1	2011	USA
MZ577038.1	2020	China
MW853953.1	2017	China
MH921912.1	2018	South Korea
NC_023020.1	2011	USA
JX896321.1	2011	USA
JX896320.1	2011	USA
MH921908.1	2017	South Korea
JX896322.1	2011	USA
MH921905.1	2017	South Korea
MW853952.1	2017	China
JX896318.1	2011	USA
MK378344.1	2017	China
MK378338.1	2017	China
MH921904.1	2017	South Korea
MW051674.1	2018	USA
MG345028.1	2017	China
MK378349.1	2017	China
MK378336.1	2017	China
MK378325.1	2017	China
MK378291.1	2017	China
MK378332.1	2017	China
MK378309.1	2017	China
MW853951.1	2017	China
MK378337.1	2017	China
MK378295.1	2017	China
MK378305.1	2017	China
MK378346.1	2017	China
MK378341.1	2017	China
MK378347.1	2017	China
MK378292.1	2017	China
MK092432.1	2015	China
MK092445.1	2015	China
MK092428.1	2015	China
MW711838.1	2018	South Korea
MW711839.1	2018	South Korea
MW711837.1	2018	South Korea
MK092442.1	2015	China
MK092440.1	2015	China
MK092426.1	2015	China
MK092438.1	2015	China
MK092434.1	2015	China
MT846938.1	2017	South Korea
MT846936.1	2017	South Korea
MK092447.1	2015	China
MK092441.1	2015	China
MK092436.1	2015	China
MK092429.1	2015	China
MK092448.1	2015	China
MK092433.1	2015	China
MK092427.1	2015	China
MK378298.1	2017	China
MK092430.1	2015	China
MK092425.1	2015	China
MK092446.1	2015	China
MK092435.1	2015	China
MK092431.1	2015	China
MK092444.1	2015	China
KU745628.1	2015	China
MK092443.1	2015	China
MK092449.1	2015	China
MK092452.1	2015	China
MK092439.1	2015	China
MK092437.1	2015	China
MK092453.1	2015	China
MK092451.1	2015	China
MK092450.1	2015	China
MK378342.1	2017	China
MN326215.1	2014	China
MN326216.1	2015	China
MN326217.1	2015	China
MN326213.1	2015	China
MN326212.1	2017	China
MN326218.1	2015	China
MN326211.1	2017	China

MN326210.1	2017	China	MK092460.1	2015	China
MN326219.1	2015	China	MK092461.1	2015	China
MN326209.1	2017	China	OR355608	2021	Colombia
MN326214.1	1999	China	OR355609	2021	Colombia
KX352458.1	2016	Poland	OR355610	2021	Colombia
KX352457.1	2016	Poland	OR355611	2021	Colombia
KX352456.1	2016	Poland	OR355612	2021	Colombia
KX352455.1	2016	Poland	OR355613	2021	Colombia
KX273436.1	2013	Poland	OR355614	2021	Colombia
KF661535.1	2013	China	OR355615	2021	Colombia
MK378326.1	2017	China	OR355616	2021	Colombia
MK378345.1	2017	China			

Table S12. Details of the 71 complete and near-complete genome (NCG) sequences of porcine parvovirus 6 (PPV6) sampled from NCBI and used in the Bayesian phylogenetic reconstruction.

Accession	Year	Country
MN326233	1998	China
MN326230	1998	China
MN326239	1999	China
MN326220	1999	China
MN326241	1999	China
KY094494	2008	Brazil
KF999682	2012	China
KF999684	2012	China
KF999685	2012	China
NC_023860	2012	China
MH558679	2012	Spain
KR709267	2014	USA
MN326232	2014	China
KR709263	2014	USA
KX273435	2014	Poland
KR709262	2014	USA
KR709264	2014	USA
KR709268	2014	USA
KR709265	2014	USA
KR709266	2014	USA
MN326240	2015	China
MN326242	2015	China
OR046033	2016	China
MN326238	2016	China
MK378356	2017	China
MK378387	2017	China
MK378405	2017	China
MZ846219	2017	China
MN326224	2017	China
MZ846222	2017	China
MZ846221	2017	China
MZ846220	2017	China
MK378369	2017	China
MK378388	2017	China
MK378361	2017	China
MK378355	2017	China

Accession	Year	Country
MK378370	2017	China
MK378381	2017	China
MK378351	2017	China
MH447540	2017	SouthKorea
MH921900	2017	SouthKorea
MH921901	2017	SouthKorea
MH447539	2017	SouthKorea
MW711841	2017	SouthKorea
MH921907	2017	SouthKorea
MH447537	2017	SouthKorea
MW711840	2017	SouthKorea
MH447535	2017	SouthKorea
MH921903	2017	SouthKorea
MK378352	2017	China
MK378373	2017	China
MZ577039	2017	China
MH447536	2017	SouthKorea
MH921909	2017	SouthKorea
MH447538	2017	SouthKorea
MH921906	2017	SouthKorea
MH447541	2017	SouthKorea
MW853955	2018	China
MW853956	2018	China
MW853954	2018	China
MW711842	2018	SouthKorea
MW853957	2018	China
MW051672	2018	USA
MH820262	2018	Canada
MH921913	2018	SouthKorea
OM811452	2019	China
OM811457	2019	China
OM811453	2019	China
OR355617	2021	Colombia
OR355618	2021	Colombia
OR355619	2021	Colombia

Table S13. List and identification of 130 PPV6-VP complete gene sequences sampled from NCBI – GenBank database and compared by phylogenetic analysis with three PPV6-VP complete sequences from Colombia (recovered from the current study).

Accession	Year	Country
**MH447538.1**	2017	South Korea
**MH921906.1**	2017	South Korea
**KR709268.1**	2014	USA
**KR709265.1**	2014	USA
**MH447541.1**	2017	South Korea
**MH558679.1**	2012	Spain
**MW051672.1**	2018	USA
**MH447536.1**	2017	South Korea
**MH921909.1**	2017	South Korea
**KX384813.1**	2016	Poland
**KX384819.1**	2016	Poland
**MH820262.1**	2018	Canada
**KY094494.1**	2008	Brazil
**KR709264.1**	2014	USA
**MW711840.1**	2017	South Korea
**KX384817.1**	2016	Poland
**KX384815.1**	2016	Poland
**MK378352.1**	2017	China
**KX384820.1**	2016	Poland
**KR709262.1**	2014	USA
**KX384821.1**	2016	Poland
**MZ577039.1**	2017	China
**KX384816.1**	2016	Poland
**MK378373.1**	2017	China
**MG760726.1**	2017	China
**MK378364.1**	2017	China
**MK378392.1**	2017	China
**MT846939.1**	2017	South Korea
**MH447535.1**	2017	South Korea
**MK378360.1**	2017	China
**MG345036.1**	2017	China
**MN326238.1**	2016	China
**MW853957.1**	2017	China
**MW711842.1**	2018	South Korea
**MT846940.1**	2017	South Korea
**MH921903.1**	2017	South Korea
**KR709263.1**	2014	USA
**KR709267.1**	2014	USA
**NC_023860.1**	2012	China
**MK378381.1**	2017	China
**MK378351.1**	2017	China
**MW853954.1**	2017	China
**MH447540.1**	2017	South Korea
**MK092462.1**	2015	China
**KX273435.1**	2014	Poland
**MW711841.1**	2017	South Korea
**MH447539.1**	2017	South Korea
**MH447537.1**	2017	South Korea
**MH921907.1**	2017	South Korea
**MH921901.1**	2017	South Korea
**KX384822.1**	2016	Poland
**MZ577040.1**	2018	China
**MN326227.1**	2017	China
**MK378355.1**	2017	China
**KX384814.1**	2016	Poland
**MK378367.1**	2017	China
**KX384818.1**	2016	Poland
**MK378386.1**	2017	China
**MK378372.1**	2017	China
**NC_075986.1**	2023	Poland
**MN326242.1**	2015	China
**MN326232.1**	2014	China
**MN326240.1**	2015	China
**MK378350.1**	2017	China
**MK378396.1**	2017	China
**MK378356.1**	2017	China
**MK378371.1**	2017	China
**MN326231.1**	1998	China
**MK378361.1**	2017	China
**MK378387.1**	2017	China
**MK378365.1**	2017	China
**MK378369.1**	2017	China
**MN326220.1**	1999	China
**MK378388.1**	2017	China
**MN326230.1**	1998	China
**MK378370.1**	2017	China
**MN326239.1**	1999	China

**MN326241.1**	1999	China	**MK092466.1**	2015	China
**MN326233.1**	1998	China	**MK092464.1**	2015	China
**KF999683.1**	2012	China	**MN326223.1**	2017	China
**MK378405.1**	2017	China	**OM811453.1**	2019	China
**MK378401.1**	2017	China	**MN326236.1**	1999	China
**MK378366.1**	2017	China	**MN326226.1**	1997	China
**MN326222.1**	2017	China	**MN326221.1**	1999	China
**MN326234.1**	2016	China	**MK378377.1**	2017	China
**OM811452.1**	2019	China	**MZ846221.1**	2017	China
**MK378368.1**	2017	China	**MN326235.1**	1999	China
**OM811456.1**	2019	China	**MN326229.1**	2017	China
**MK092467.1**	2015	China	**OM811449.1**	2019	China
**MK378399.1**	2017	China	**MW853955.1**	2017	China
**KF999682.1**	2012	China	**MN326224.1**	2017	China
**KF999681.1**	2013	China	**MN326225.1**	2011	China
**MK378376.1**	2017	China	**MK378389.1**	2017	China
**OM811454.1**	2019	China	**MK378353.1**	2017	China
**MZ846219.1**	2017	China	**MK378354.1**	2017	China
**KF999684.1**	2012	China	**MZ491196.1**	2018	South Korea
**MK092465.1**	2015	China	**MZ491197.1**	2018	South Korea
**OM811455.1**	2019	China	**MZ491194.1**	2018	South Korea
**MK092463.1**	2015	China	**MK092475.1**	2015	China
**MZ846220.1**	2017	China	**MK092476.1**	2015	China
**MZ846222.1**	2017	China	**OR355617**	2021	Colombia
**OM811457.1**	2019	China	**OR355618**	2021	Colombia
**MW853956.1**	2017	China	**OR355619**	2021	Colombia
**MN326228.1**	1999	China			

Table S14. Details of the 49 complete and near-complete genome (NCG) sequences of porcine parvovirus 7 (PPV7) sampled from NCBI and used in the Bayesian phylogenetic reconstruction.

Accession	Year	Country
MN326262	1997	China
MN326273	1998	China
MN326274	1998	China
MN326275	1998	China
MN326251	1998	China
MN326266	2011	China
MN326268	2011	China
MK092491	2015	China
MK092483	2015	China
MK092490	2015	China
MK092486	2015	China
MK092479	2015	China
MK092478	2015	China
MG543462	2015	China
MG543467	2015	China
MG543466	2015	China
MG543472	2015	China
MG543461	2015	China
MG543465	2015	China
MG543470	2015	China
MK092482	2015	China
MK092494	2015	China
MG543457	2015	China
MG543456	2015	China
MG543458	2015	China

Accession	Year	Country
MG543469	2015	China
MG543468	2015	China
MG543460	2015	China
MG543463	2015	China
MG543464	2015	China
MN326258	2017	China
MZ846211	2017	China
MZ846210	2017	China
MN326261	2017	China
MN326257	2017	China
MN326263	2017	China
MN326264	2017	China
MN326260	2017	China
MK484101	2018	China
MW853959	2018	China
MW853960	2018	China
MW853961	2018	China
MZ577044	2018	China
MZ577043	2018	China
ON462335	2021	China
OR349220	2021	Colombia
OR349221	2021	Colombia
ON462331	2021	China
ON462333	2021	China

Table S15. List and identification of 185 PPV7-VP complete gene sequences sampled from NCBI – GenBank database and compared by phylogenetic analysis with two PPV7-VP complete sequences from Colombia (recovered from the current study).

Accession	Year	Country
**MH422967.1**	2017	South Korea
**KU563733.1**	2015	USA
**MT747168.1**	2015	Colombia
**MK092492.1**	2015	China
**MW853961.1**	2017	China
**MT758695.1**	2019	Colombia
**MK092489.1**	2015	China
**MT758697.1**	2015	Colombia
**MK092488.1**	2015	China
**MH422966.1**	2017	South Korea
**MK092484.1**	2015	China
**MK092482.1**	2015	China
**MH422962.1**	2017	South Korea
**MT758696.1**	2015	Colombia
**MN326254.1**	2014	China
**MN326257.1**	2017	China
**MG543470.1**	2015	China
**MK092493.1**	2015	China
**MG543472.1**	2015	China
**MK092481.1**	2015	China
**MH422964.1**	2017	South Korea
**NC_040562.1**	2015	China
**MK484102.1**	2018	China
**MW051670.1**	2019	USA
**MG543457.1**	2015	China
**MG543458.1**	2015	China
**MH422965.1**	2017	South Korea
**MH422963.1**	2017	South Korea
**MN326268.1**	2011	China
**MK092486.1**	2015	China
**MN326256.1**	2011	China
**MG543469.1**	2015	China
**MN326255.1**	2015	China
**MZ846213.1**	2017	China
**MG543456.1**	2015	China
**MG543460.1**	2015	China
**MK484100.1**	2018	China
**MK092487.1**	2015	China
**MK092491.1**	2015	China
**MW916962.1**	2021	China
**MG543467.1**	2015	China
**MN326278.1**	2014	China
**MN326260.1**	2017	China
**MN326272.1**	1998	China
**MH817776.1**	2018	South Korea
**MN326277.1**	1998	China
**MH817777.1**	2018	South Korea
**MN326270.1**	2011	China
**MN326274.1**	1998	China
**MN326280.1**	1998	China
**MN326267.1**	2011	China
**MN326275.1**	1998	China
**OL856077.1**	2021	China
**OL856076.1**	2021	China
**MN326273.1**	1998	China
**MN326250.1**	2011	China
**MN326247.1**	2017	China
**MN326245.1**	2017	China
**MZ803107.1**	2017	China
**MN326282.1**	2017	China
**KY996757.1**	2017	China
**ON462332.1**	2021	China
**MN326248.1**	2011	China
**MG914435.1**	2016	Sweden
**MN326252.1**	1999	China
**MK092480.1**	2015	China
**KY996758.1**	2014	China
**MN326244.1**	1999	China
**MN326246.1**	1999	China
**MN326276.1**	2017	China
**MN326243.1**	1999	China
**MK092485.1**	2015	China
**MN326283.1**	1999	China
**MN326271.1**	1999	China
**MN326259.1**	2011	China
**MN326289.1**	2016	China

**MG543459.1**	2015	China	**MK092477.1**	2015	China
**MK092490.1**	2015	China	**ON462335.1**	2021	China
**MT846941.1**	2017	South Korea	**MN326292.1**	1998	China
**MG696111.1**	2017	China	**MN326258.1**	2017	China
**ON462337.1**	2021	China	**MN326253.1**	1998	China
**MG543465.1**	2015	China	**MN326290.1**	2014	China
**MG543463.1**	2015	China	**MN326291.1**	2017	China
**MK092494.1**	2015	China	**MK092495.1**	2015	China
**MG543464.1**	2015	China	**MN326287.1**	1999	China
**MZ846210.1**	2017	China	**MN326288.1**	1999	China
**MG696112.1**	2017	China	**MN326293.1**	2017	China
**MG902949.1**	2016	China	**MN326286.1**	1999	China
**MW853962.1**	2017	China	**MK378415.1**	2017	China
**MG543468.1**	2015	China	**MZ803101.1**	2017	China
**MG543466.1**	2015	China	**ON568788.1**	2021	China
**MG543461.1**	2015	China	**MH293509.1**	2018	South Korea
**MZ846212.1**	2017	China	**ON568789.1**	2021	China
**MN326281.1**	2017	China	**ON568793.1**	2022	China
**MW853960.1**	2017	China	**ON568785.1**	2021	China
**ON462331.1**	2021	China	**MZ846215.1**	2018	China
**MN326251.1**	1998	China	**MZ803102.1**	2017	China
**MW853959.1**	2017	China	**ON568787.1**	2021	China
**MZ846211.1**	2017	China	**MZ846216.1**	2018	China
**OL856075.1**	2021	China	**MZ803099.1**	2017	China
**MW051671.1**	2018	USA	**MZ803098.1**	2017	China
**MN515032.1**	2016	Brazil	**MZ846214.1**	2018	China
**MZ577044.1**	2018	China	**ON568790.1**	2021	China
**MK092479.1**	2015	China	**ON568784.1**	2021	China
**MN326285.1**	2015	China	**MZ491199.1**	2019	South Korea
**ON462336.1**	2021	China	**ON568791.1**	2021	China
**MK092478.1**	2015	China	**MZ803103.1**	2016	China
**MZ577045.1**	2019	China	**MZ803093.1**	2017	China
**ON462333.1**	2021	China	**ON568786.1**	2021	China
**MZ846209.1**	2017	China	**MZ491200.1**	2019	China
**KY996756.1**	2014	China	**MZ803097.1**	2017	China
**MZ577042.1**	2017	China	**MZ803092.1**	2017	China
**MG543462.1**	2015	China	**MZ491198.1**	2019	South Korea
**OL856078.1**	2021	China	**MZ803094.1**	2017	China
**MK484101.1**	2018	China	**MZ803091.1**	2017	China
**MZ577043.1**	2018	China	**MZ803089.1**	2017	China
**MK092483.1**	2015	China	**ON568794.1**	2022	China
**ON462334.1**	2021	China	**MZ803105.1**	2016	China
**MN326266.1**	2011	China	**MZ803096.1**	2017	China

**MN326265.1**	2017	China	**MH293508.1**	2018	South Korea
**MK378411.1**	2017	China	**ON568792.1**	2022	China
**MN326269.1**	2011	China	**MZ803095.1**	2017	China
**MN326261.1**	2017	China	**MZ803104.1**	2016	China
**MN326284.1**	2015	China	**MH293507.1**	2018	South Korea
**MW853958.1**	2017	China	**MZ803100.1**	2017	China
**MN326263.1**	2017	China	**MZ803090.1**	2017	China
**MK092496.1**	2015	China	**MZ803106.1**	2016	China
**MN326279.1**	1999	China	**OR349220**	2021	Colombia
**MN326264.1**	2015	China	**OR349221**	2021	Colombia
**MN326262.1**	1997	China	**MZ577041.1**	2017	China
**MN326249.1**	2011	China			

Table S16. Primers used for sequencing porcine parvoviruses (PPV1 through PPV7)

Primer	Sequence 5`-3`	Length	Genbank position/Accession number	Reference
**PPV1 S1F**	ACCAACCTGCACTTAACTCC	970	2030-3000 GQ884042 a	[6]
**PPV1 S1R**	GTGTGTGTGCATCGTCTTGT			
**PPV1 S2F**	GAGGTAAGAAGATCGCCGAG	1136	2562-3698 GQ884042 a	
**PPV1 S2R**	TCCTACCTGAGCTGGCCTAA			
**PPV1 S3F**	CTACCACAGAAGGAGACCAA	928	3570-4498 GQ884042 a	
**PPV1S3R**	ATTGAAGTATACAATGATAGTAGT			
**PPV2S1Ry**	CCGACAGGATAAGTGTCGAG	1246	151-1397/MW853943b	[17]
**PPV2 1Q2**	GTTTGCCCTTAATGTCGATCC			
**PPV2 S2F**	CCTCTCAATCCCTGATTTGG	1137	1340-2477/MW853943b	This study
**PPV2 S2R**	GTAAAGCTGTAACAGAGCCTGCC			
**PPV2 S3F**	GGATCAATGCCCTTCTGAGG	973	2361-3334/MW853943b	
**PPV2 S3R**	GGTAAGACTTTGTCCAGCTCC			
**PPV2 S4F**	GGACACGTTAGCGGATCTCAT	1003	3340-4343/MW853943b	
**PPV2 S4R**	GGTTATGACGGAGGTTCAACG			
**PPV2-S5F**	TTACGAGTTTCCCAGTCTCG	1150	4273-5423/MW853943b	[17]
**PPV2-S5R**	CCGACAGGATAAGTGTCGAG			
**PPV3-S1F**	GTTGCGTATTTCCGCTTCC	785	147-932/MK378244c	This study
**PPV3 S1R**	CTTATCCGTGGCCAACCAC			
**PPV3 S2F**	GCATAGCCACCGAACGAC	940	895-1835/MK378244 c	
**PPV3 S2R**	CTCGGAGACAGACACGTC			
**PPV3 S3 F**	GATGAGTCCACGGACGTG	932	1806-2738/MK378244 c	
**PPV3 S3 R**	CAAGGAGAGACACTCTCCTC			
**PPV3 S4 F**	GATGCCGTAGTACCTTCTGC	951	2677-3628/MK378244 c	
**PPV3 S4 R**	GCAGCTGCTGGAAATCC			
**PPV3 S5 F**	GGCAGCCTGTTATGGGTATG	1500	3536-5131/MK378244 c	
**PPV3 S5 R**	GGAAGATGAGCTGGCCTTG			
**PPV4 S1F**	TGACGCAGTACAGACCGACGAGA	841	356-1197/ MW073110d	[89]
**PPV4 S1R**	AATGCAAGTGCAAGCCACCTTTT			
**PPV4 S2F**	AGTAATCTGGTAATCGCTGTTCG	892	1050-1942/ MW073110d	
**PPV4 S2R**	ATGTTAGTCTTTCCTGTTGTGGC			
**PPV4 S3F**	GCTGGTGGATAACAACATCTGCT	985	1568-2553/ MW073110d	
**PPV4 S3R**	GTTTCTTCTTTCTCGGTGCTTCT			
**PPV4 S4F**	AAGAAGCACCGAGAAAGAAGAAA	883	2530-3413/ MW073110d	
**PPV4 S4R**	AAATCTAAGGGACAAGGCAAACG			
**PPV4 S5F**	AGATACTAAGAAAGACAAGGTGGAG	900	3363-4263/ MW073110d	
**PPV4 S5R**	AATAATAGAAGGTATAGCGTCTCCA			
**PPV4 S6F**	ACCTGCTCCTCCATCTTCTCCAC	979	3918-4897/ MW073110d	
**PPV4 S6R**	GGCCGTCATCATACATTCTGCTC			
**PPV4 S7F**	ACTTACTGTTCTATGATGTCTGGAG	1643	4219-5862/ MW073110d	
**PPV4 S7R**	ATATCATCTGCGGTGTCTGGG			
**PPV5 S1F**	TGACGCAGTACAGACCGACGAGA	793	146-1149/MW051674e	[90]
**PPV5 S1R**	CGCCAGACTCACAGTTTTCATT			
**PPV5 S2F**	GGGATGTGACGCAGTACAGACC	1222	482-1704/MW051674e	
**PPV5 S2R**	CCAACCACTGTACAATGTCTAGCAT			
**PPV5 S3F**	CCATGTACAAATGTGCCACTG	1104	1651-2755/MW051674e	
**PPV5 S3R**	GGAACAGGTTCAAAGTTGTCATC			
**PPV5 S4F**	CAAACATGGAGCGGGAGAAGAC	975	3601-4576/MW051674e	
**PPV5 S4R**	GAAGATGACCAGGCACTGTTAGCTG			
**PPV5 S5F**	GTTTTGAGGATACACATTACGAGC	827	4554-5381/MW051674e	
**PPV5 S5R**	GAG AAG GCA TTC CAG GTG TG			
**PPV5 S6F**	GTT ACA GGG TAC AGG CTT GCT G	559	5318-5877/MW051674e	
**PPV5 S6R**	CTT CTC GCT CTA ACA CGT TGC			
**PPV6 S1F**	CGAAAGCCTCTGTATTATGTCT	1088	203-1291/ MG760726 f	This study
**PPV6 S1R**	TTGCGTTATTCCAGTTAACAC			
**PPV6 S2F**	TGTTTGGACCTGCTACAACCG	1265	1219-2484/ MG760726 f	
**PPV6 S2R**	TTAAAACCCTGAACACCGGAA			
**PPV6 S3F**	CTCTCAGTCAATCCGACGTT	1494	2113-3607/ MG760726 f	
**PPV6 S3R**	ATCTCGCTGAGCATTATCCAA			
**PPV6 S5F**	TCTTAAACATGGAGACTGGC	1090	3567-4654/ MG760726 f	
**PPV6 S5R**	CCTCGAATAAGAGTGGCATC			
**PPV6 S6F**	CAGTCAGCCACCATTGCTA	1395	4423-5818/ MG760726 f	
**PPV6 S6R**	TCAACCAGAATGCCTGACAC			
**PPV7 S1F**	GTGGTGGGGCTGGCTGAGCTG	977	351-1326/ MN326293g	[42]
**PPV7 S1R**	GCGTTGTCTTGCATGGACCAG			

**PPV7 S2F**	ACACAAGCCGGGATTCCAGCA	1013	1270-2252/ MN326293[g]	
**PPV7 S2R**	CCACGAGCACTCCATCCCCTC			
**PPV7 S3F**	CGCAAGACTTGGCTTCAGCAC	1066	2128-3193/ MN326293[g]	
**PPV7 S3R**	GGATGCTGTCCGGGTTGGTGA			
**PPV7 S4F**	AGCTGGAGCAGACTTACACTC	1032	2992-4024/ MN326293 [g]	
**PPV7 S4R**	GCGGGTTAATAACAGGAAGGG			

[a,b,c,d,e,f,g] GenBank reference sequence of each PPVs. Primers PPV2 S2-S4, PPV3 S1-S6 and PPV6 S1-S6 were designed.

Table S17. Sequencing coverage of each porcine parvovirus (PPVs).

PPV	Coverage of each genome (nt)	Percentage of coverage	Reference strain
PPV1	2190	44.2	U44978
PPV2	5269	96	GU938300
PPV3	5091	99.5	KY586145
PPV4	5507	93.2	NW073110
PPV5	5395	93.6	MW853953
PPV6	5606	90.4	KY094494
PPV7	3703	92	MG902949

Table S18. List and identification of 31 Colombian PPVs genomes obtained in the current study and used for phylogenetic analyses

No.	Name	Region	Virus	GenBank no.
1	PPV1/Colombia112/2021	Antioquia	PPV1	PP921711
2	PPV1/Colombia114/2021	Antioquia	PPV1	PP921712
3	PPV2/Colombia131/2021	Antioquia	PPV2	OR349215
4	PPV2/Colombia275/2021	Cundinamarca	PPV2	OR349216
5	PPV2/Colombia433/2021	Eje cafetero	PPV2	OR349217
6	PPV2/Colombia463/2021	Eje cafetero	PPV2	OR349218
7	PPV2/Colombia471/2021	Eje cafetero	PPV2	OR349219
8	PPV3/Colombia112/2021	Antioquia	PPV3	OR349222
9	PPV3/Colombia125/2021	Antioquia	PPV3	OR349223
10	PPV3/Colombia315/2021	Atlántico	PPV3	OR349224
11	PPV3/Colombia336/2021	Atlántico	PPV3	OR349225
12	PPV3/Colombia345/2021	Atlántico	PPV3	OR349226
13	PPV3/Colombia353/2021	Atlántico	PPV3	OR349227
14	PPV4/Colombia112/2021	Antioquia	PPV4	OR349292
15	PPV4/Colombia252/2021	Antioquia	PPV4	OR349293
16	PPV4/Colombia315/2021	Atlántico	PPV4	OR349294
17	PPV4/Colombia575/2021	Valle	PPV4	OR349295
18	PPV5/Colombia162/2021	Antioquia	PPV5	OR355608
19	PPV5/Colombia184/2021	Antioquia	PPV5	OR355609
20	PPV5/Colombia193/2021	Antioquia	PPV5	OR355610
21	PPV5/Colombia222/2021	Cundinamarca	PPV5	OR355611
22	PPV5/Colombia326/2021	Atlántico	PPV5	OR355612
23	PPV5/Colombia354/2021	Atlántico	PPV5	OR355613
24	PPV5/Colombia511/2021	Valle	PPV5	OR355614
25	PPV5/Colombia532/2021	Valle	PPV5	OR355615
26	PPV5/Colombia553/2021	valle	PPV5	OR355616
27	PPV6/Colombia255/2021	Cundinamarca	PPV6	OR355617
28	PPV6/Colombia563/2021	Valle	PPV6	OR355618
29	PPV6/Colombia572/2021	Valle	PPV6	OR355619
30	PPV7/Colombia3/2021	Atlántico	PPV7	OR349220
31	PPV7/Colombia581/2021	Valle	PPV7	OR349221

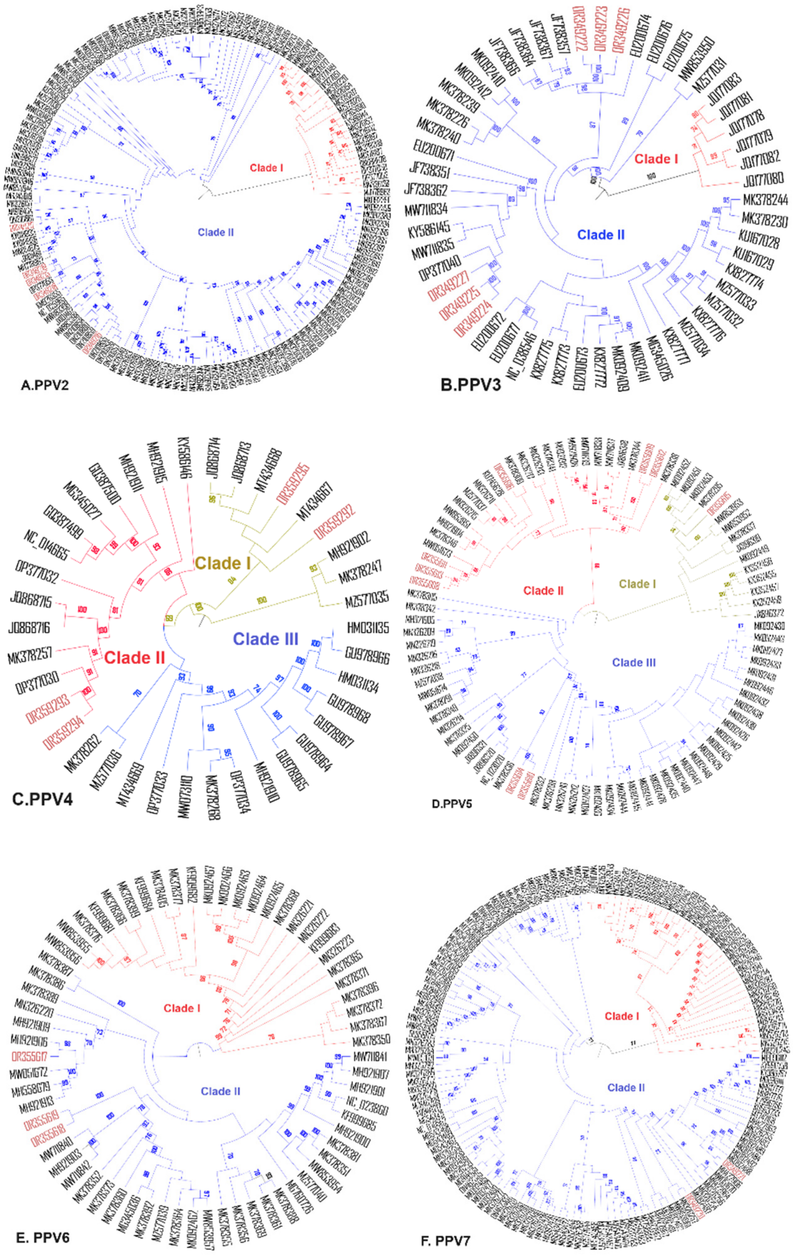

Figure S1. Maximum likelihood phylogenetic trees of Porcine parvoviruses (PPVs) inferred from the alignment of NCG nucleotides: A. PPV2, B. PPV3, C. PPV4, D. PPV5, E. PPV6, and F. PPV7. Colombian sequences are highlighted in red.

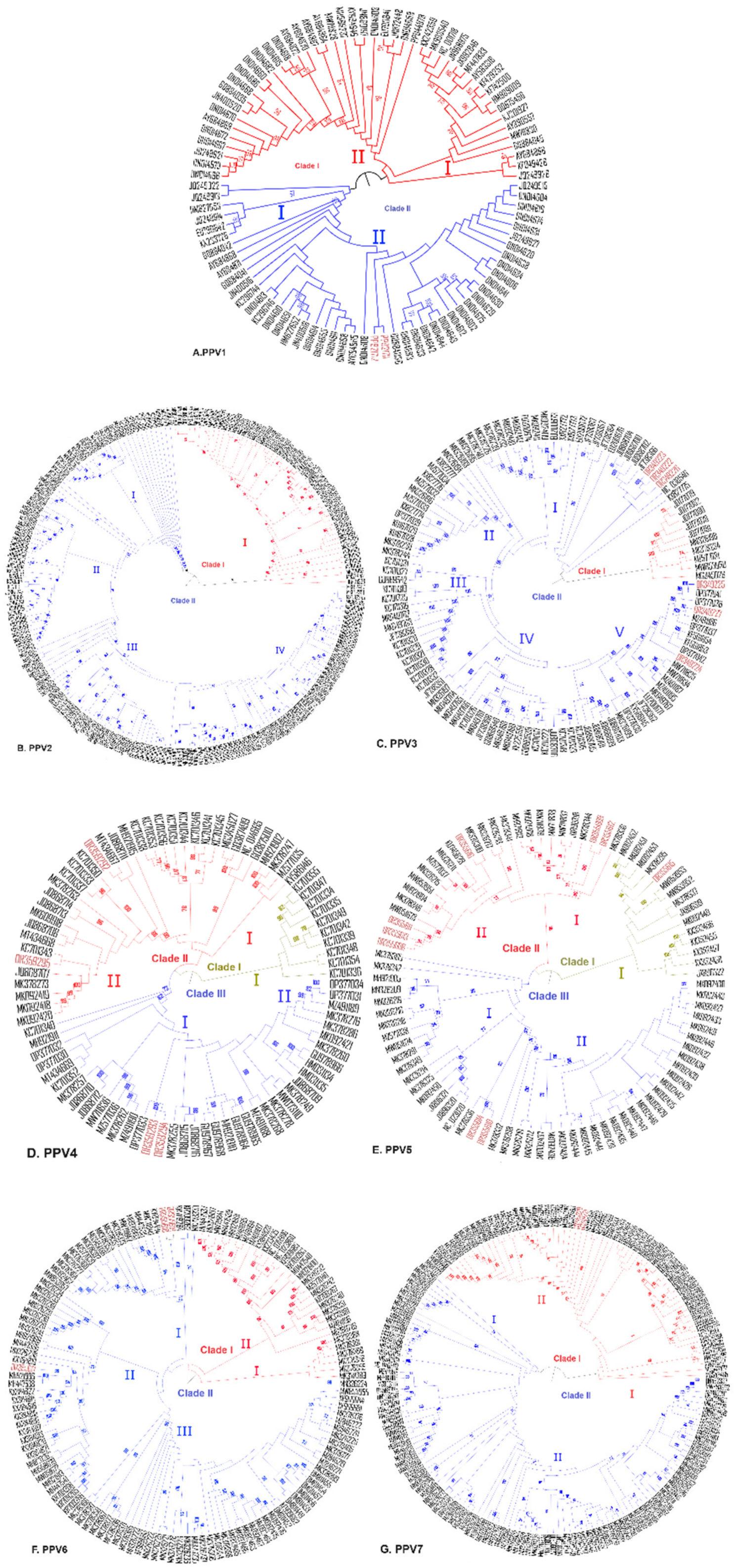
A.PPV1
Clade I
Clade II
B. PPV2
Clade I
Clade II
C. PPV3
Clade I
Clade II
D. PPV4
Clade I
Clade II
Clade III
E. PPV5
Clade I
Clade II
Clade III
F. PPV6
Clade I
Clade II
G. PPV7
Clade I
Clade II

Figure S2. Maximum likelihood phylogenetic trees of Porcine parvoviruses (PPVs) inferred from the alignment of VP nucleotides with statistical support. A. PPV1, B. PPV2, C. PPV3, D. PPV4, E. PPV5, F. PPV6, and G. PPV7. Colombian sequences are highlighted in red.

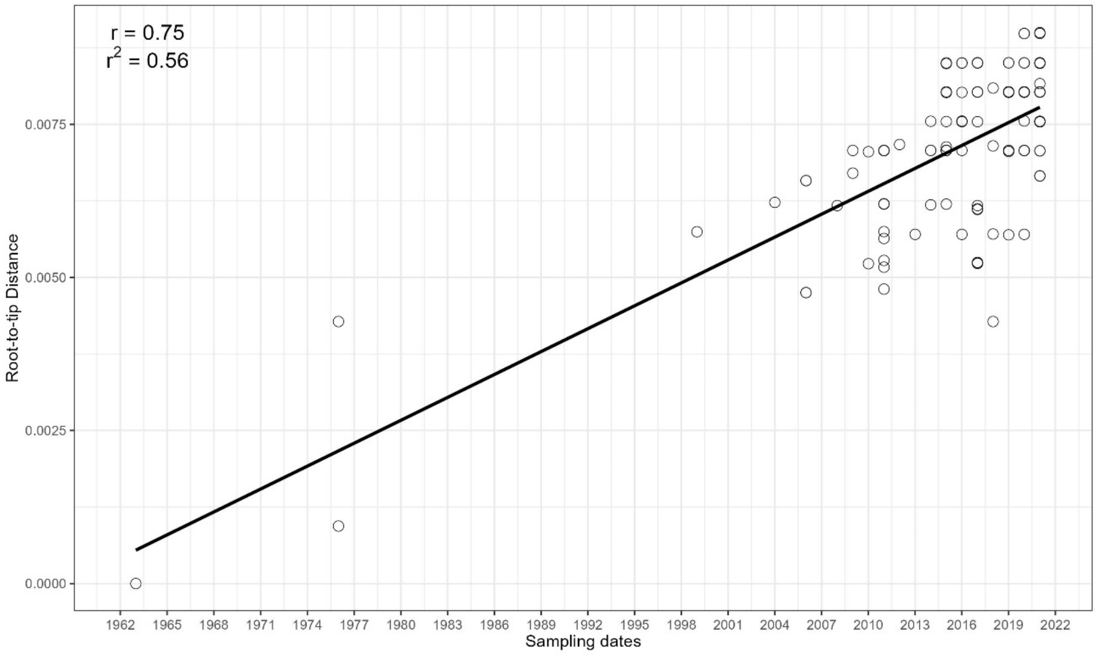

Figure S3. Root-to-tip regression of genetic distances against sampling dates for 114 Porcine parvovirus 1 (PPV1) VP1/2/3 sequences in the final dataset. The correlation coefficient (r) and r-squared (r^2) values are shown above the graph.

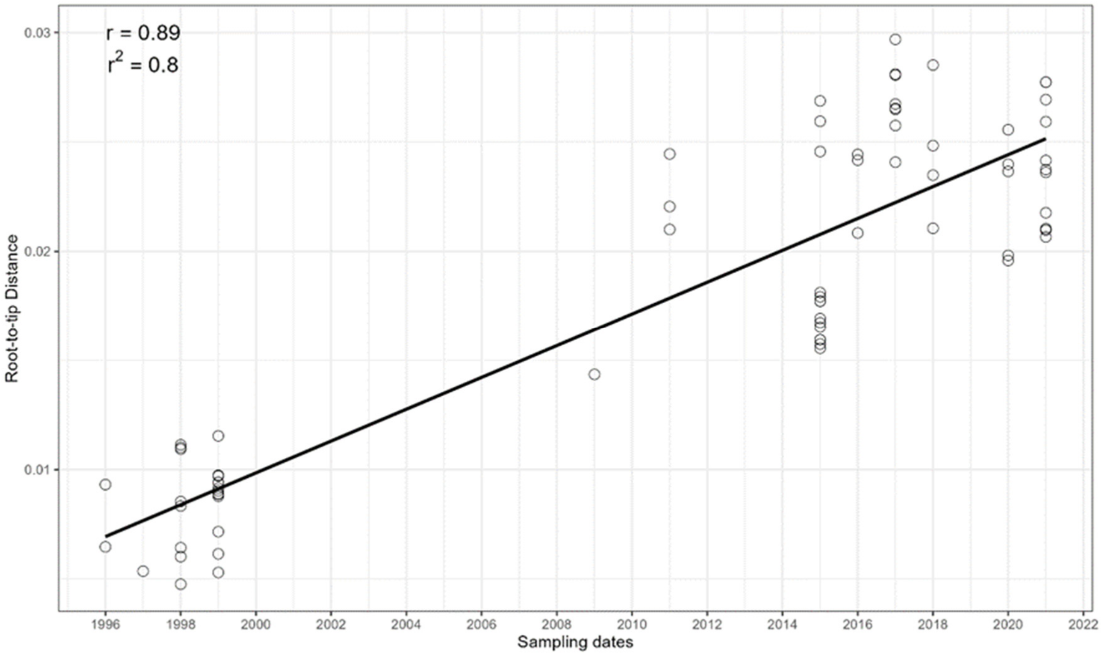

Figure S4. Root-to-tip regression of genetic distances against sampling dates for 74 Porcine parvovirus 2 (PPV2) genomes in the final dataset. The correlation coefficient (r) and r-squared (r^2) values are shown above the graph.

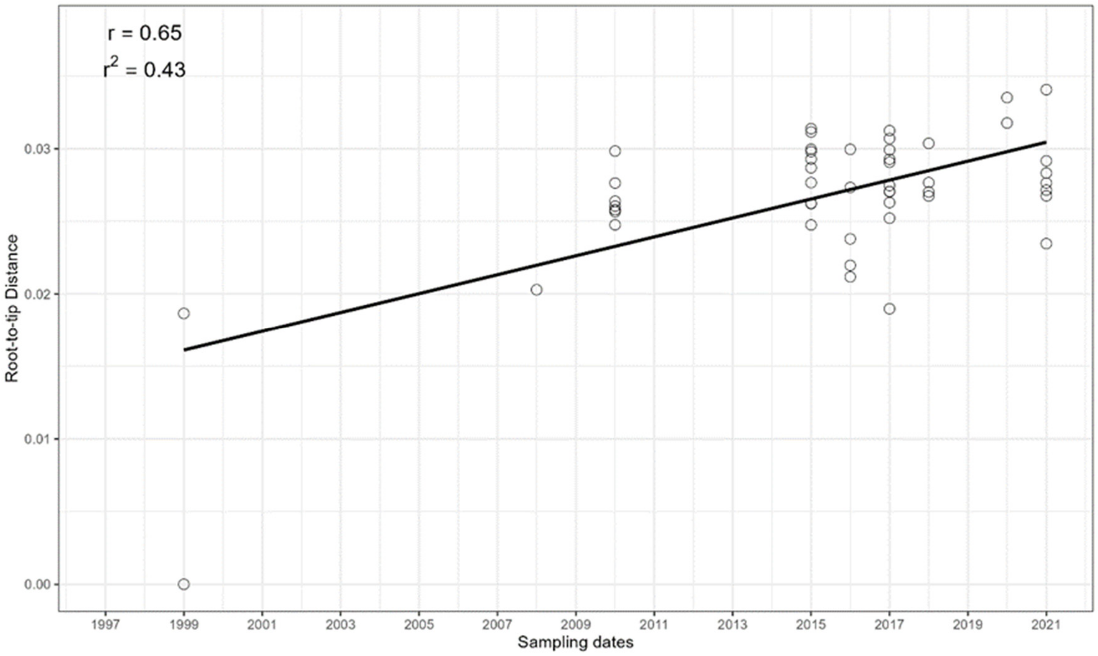

Figure S5. Root-to-tip regression of genetic distances against sampling dates for 52 Porcine parvovirus 3 (PPV3) genomes in the final dataset. The correlation coefficient (r) and r-squared (r^2) values are shown above the graph.

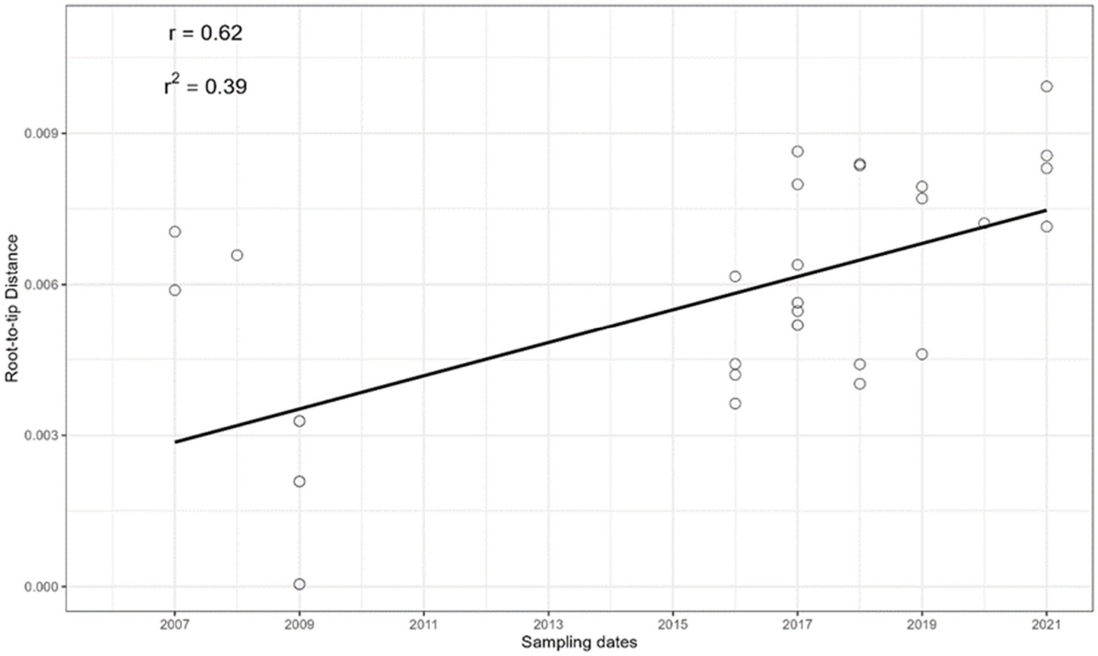

Figure S6. Root-to-tip regression of genetic distances against sampling dates for 32 Porcine parvovirus 4 (PPV4) genomes in the final dataset. The correlation coefficient (r) and r-squared (r^2) values are shown above the graph.

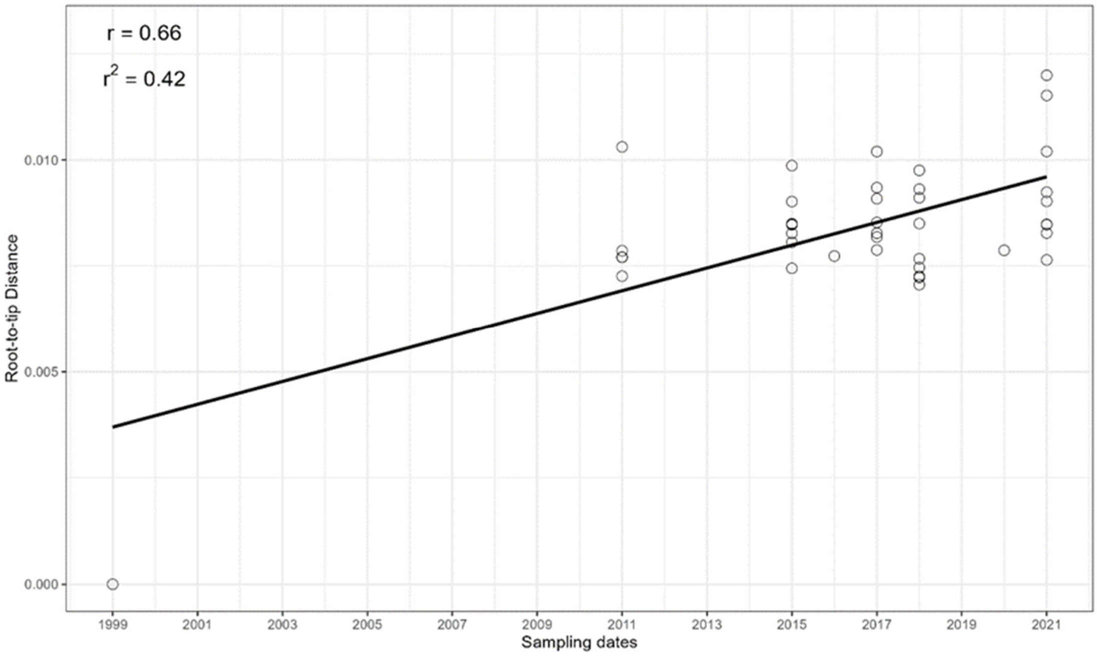

Figure S7. Root-to-tip regression of genetic distances against sampling dates for 42 Porcine parvovirus 5 (PPV5) genomes in the final dataset. The correlation coefficient (r) and r-squared (r²) values are shown above the graph.

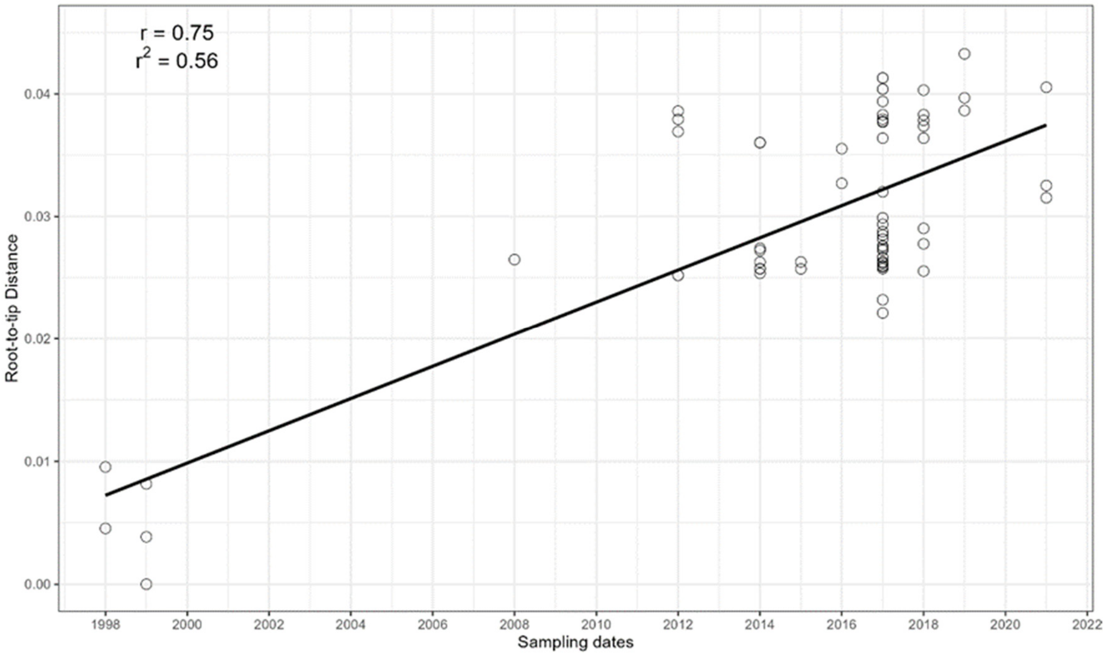

Figure S8. Root-to-tip regression of genetic distances against sampling dates for 71 Porcine parvovirus 6 (PPV6) genomes in the final dataset. The correlation coefficient (r) and r-squared (r²) values are shown above the graph.

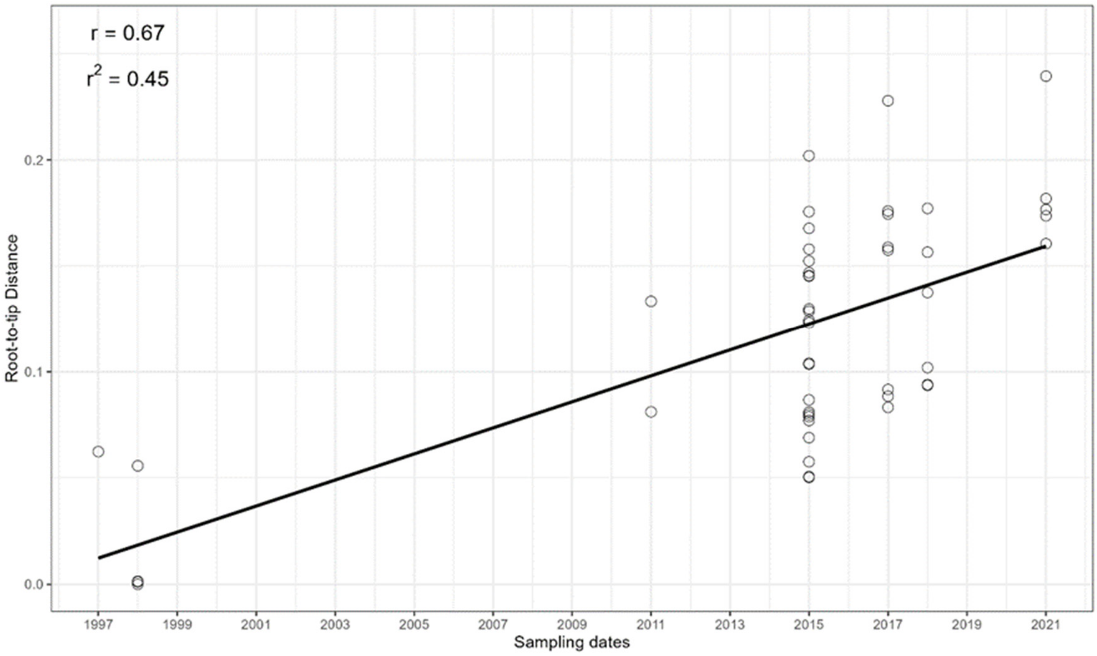

Figure S9. Root-to-tip regression of genetic distances against sampling dates for 49 Porcine parvovirus 7 (PPV7) genomes in the final dataset. The correlation coefficient (r) and r-squared (r^2) values are shown above the graph.
